# The sky compass network in the brain of the desert locust

**DOI:** 10.1007/s00359-022-01601-x

**Published:** 2022-12-23

**Authors:** Uwe Homberg, Ronja Hensgen, Stefanie Jahn, Uta Pegel, Naomi Takahashi, Frederick Zittrell, Keram Pfeiffer

**Affiliations:** 1 Department of Biology, Animal Physiology & Center for Mind Brain and Behavior (CMBB), Philipps-University of Marburg and Justus Liebig University of Giessen, 35032 Marburg, Germany; 2 Behavioral Physiology and Sociobiology (Zoology II), Biocenter, University of Würzburg, 97074 Würzburg, Germany

**Keywords:** Polarization vision, Central complex, Sky compass coding, Intracellular recordings, Desert locust

## Abstract

Many arthropods and vertebrates use celestial signals such as the position of the sun during the day or stars at night as compass cues for spatial orientation. The neural network underlying sky compass coding in the brain has been studied in great detail in the desert locust *Schistocerca gregaria*. These insects perform long-range migrations in Northern Africa and the Middle East following seasonal changes in rainfall. Highly specialized photoreceptors in a dorsal rim area of their compound eyes are sensitive to the polarization of the sky, generated by scattered sunlight. These signals are combined with direct information on the sun position in the optic lobe and anterior optic tubercle and converge from both eyes in a midline crossing brain structure, the central complex. Here, head direction coding is achieved by a compass-like arrangement of columns signaling solar azimuth through a 360° range of space by combining direct brightness cues from the sun with polarization cues matching the polarization pattern of the sky. Other directional cues derived from wind direction and internal self-rotation input are likely integrated. Signals are transmitted as coherent steering commands to descending neurons for directional control of locomotion and flight.

## Introduction

**Fig. 1:**
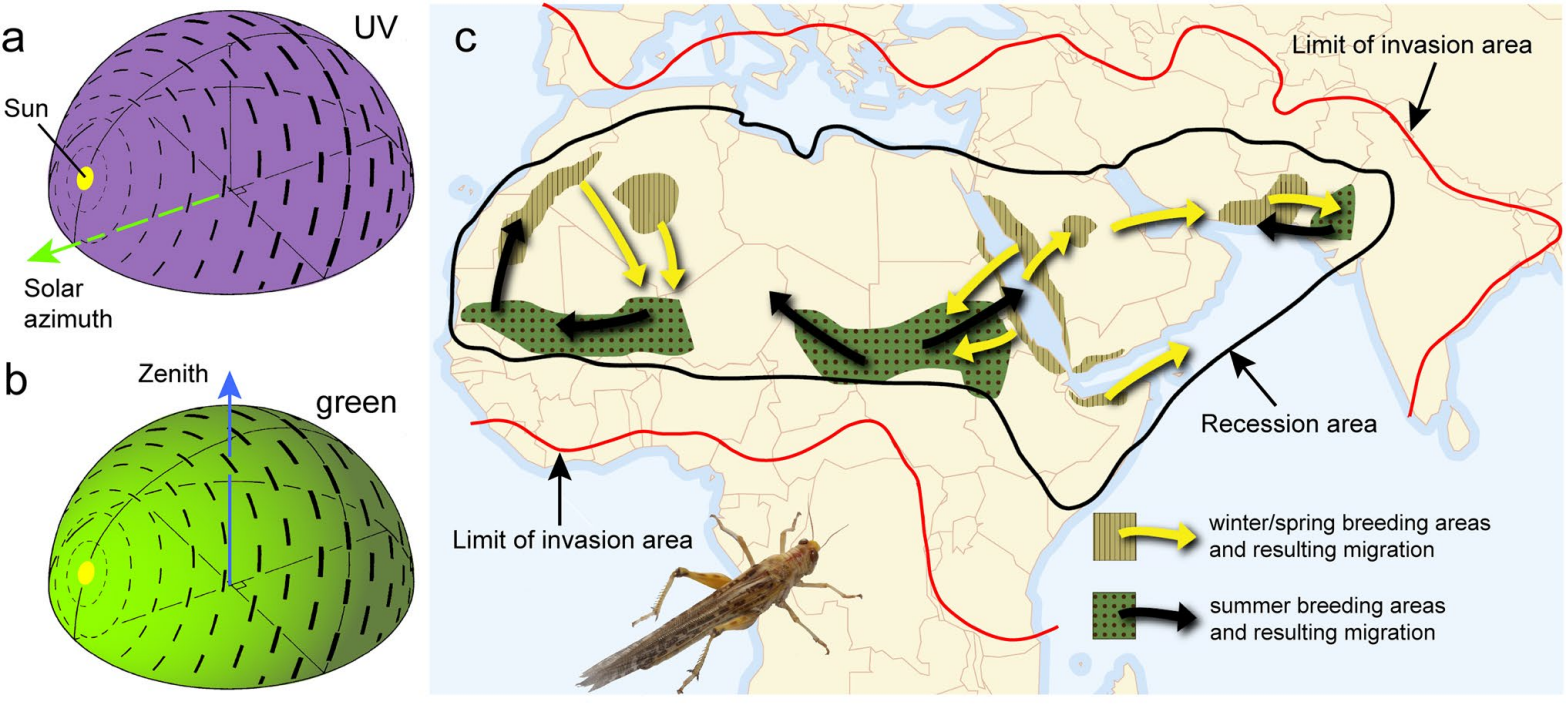
**a, b** Compass signals of the daytime sky include the position of the sun, the chromatic gradient between short-wavelength light (UV) with uniform intensity across the sky (**a**) and long-wavelength light (green) with high intensity in the solar hemisphere and lower intensity in the anti-solar hemisphere of the sky (**b**), and the polarization pattern of the sky (**a,b**). Bar orientation indicates the angle of polarization (AoP). While direct sunlight is unpolarized, the degree of polarization, indicated by the thickness of the bars, increases with increasing angular distance from the sun. **c** Recession and invasion areas of the desert locust *S. gregaria*. **a,b** Modified from el Jundi et al. (2014), **c** modified from Cressman (2016) with permission from the publisher

Many animals, ranging from sandhoppers to birds and humans, use celestial cues to determine and maintain directions during short-range spatial orientation and long-range migration (Wehner 1984; Ugolini et al. 2016; Foster et al. 2018; Mouritsen 2000). This allows animals to maintain a chosen angle of locomotion relative to sky compass signals. These can be celestial bodies like the sun, the stars, the milky way, or the moon, the chromatic gradient or polarization pattern of the sky, as well as various combinations of these. Although the salience of these cues strongly depends on clouding conditions, some of these factors may still be detected under a fully overcast sky (Coulson 1988; Hegedüs et al. 2007). Unlike vertebrates, arthropods possess microvillar photoreceptors that are inherently sensitive to the polarization angle of light (Roberts et al. 2011; Homberg and el Jundi 2013) and are, therefore, particularly suited for the detection and use of sky polarization as a navigational cue. The polarization pattern is generated by the sun through the scattering of sunlight in the atmosphere (Rayleigh scattering; Strutt 1871). It is characterized by systematic changes in the degree and angle of polarization (Fig. 1a,b). Because this pattern is tightly linked to the position of the sun, it moves with the sun during its daily course. The degree of polarization ranges from zero (direct sunlight) to a maximum of about 0.75 (75%) at an angle of 90° away from the sun even under the most favorable conditions (cloudless blue sky). The pattern of sky polarization is relatively robust against haze or various clouding conditions, but the degree of polarization is more strongly affected and is reduced to a maximum of 0.15 (15%) under fully overcast skies (Brines and Gould 1982; Hegedüs et al. 2007). Because polarization of light is exploited by land animals only in specific other situations, e.g. to facilitate object detection or the surface of bodies of water, it is an ideal property of light to be used by a “private” sensory channel for navigational purposes. Insects make particular use of this. Many insects have devoted a specifically adapted dorsal eye region, the dorsal rim area (DRA) to the detection of sky polarization (Labhart and Meyer 1999). This opens a window to explore neural mechanisms in the brain involved in the processing of celestial polarization signals and, finally, allowed identifying and characterizing brain areas involved in the coding of heading directions (Homberg et al. 2011; Hardcastle et al. 2021). Using this strategy, the processing of sky compass signals in the insect brain has been investigated in great detail in the desert locust *Schistocerca gregaria* (Homberg et al. 2011; Zittrell et al. 2020; Takahashi et al. 2022).

Desert locusts are considered one of the most dangerous pest species in Northern Africa and the Middle East (Cressman 2016). A key feature of their lifestyle is a prominent phase polyphenism, a density-dependent change between a cryptic, solitarious phenotype at low density, and a swarm-forming gregarious phenotype at high density of individuals. Both phases differ in coloration, form and behavior, and daily activity patterns (Pener and Simpson 2009). In the solitarious phase, locusts become active around sunset. They largely disperse locally and only occasionally cover distances of over 10 to 100 km per night (Farrow 1990). Heavy rainfall leads to an increase in locust population and, once the desert starts to dry out again, locusts aggregate, form hopper bands and eventually flying swarms that migrate during the day. In both phases, migrations roughly follow seasonal changes in rainfall within the intertropical convergence zone resulting in back-and-forth or circular migrations between winter/spring and summer breeding areas (Fig. 1c; Farrow 1990; Homberg 2015; Cressman 2016). Adult locusts are largely passive fliers, i.e. they are carried by the wind, but at low wind speed flight tracks become largely independent of wind direction (Farrow 1990; Roffey 1963). Frequent observations of flight courses that significantly deviate from prevailing wind directions indicate that active orientation underlies at least partly locust migrations (Kennedy 1951; Baker et al. 1984; Farrow 1990). By experimentally manipulating the sun position through a mirror, Kennedy (1945, 1951) demonstrated that a sun compass plays a significant role in the maintenance of direction of marching hopper bands and flying adults. Interestingly, diverting the animals by an artificial sun from their original course only worked in isolated animals at the edge of a hopper band and not among densely crowded hoppers suggesting that both the strong tendency to follow the common direction and a sun compass determines the stability of navigation direction in unobstructed terrain.

## The locust eye: photoreceptors sensitive to sky compass signals

**Fig. 2:**
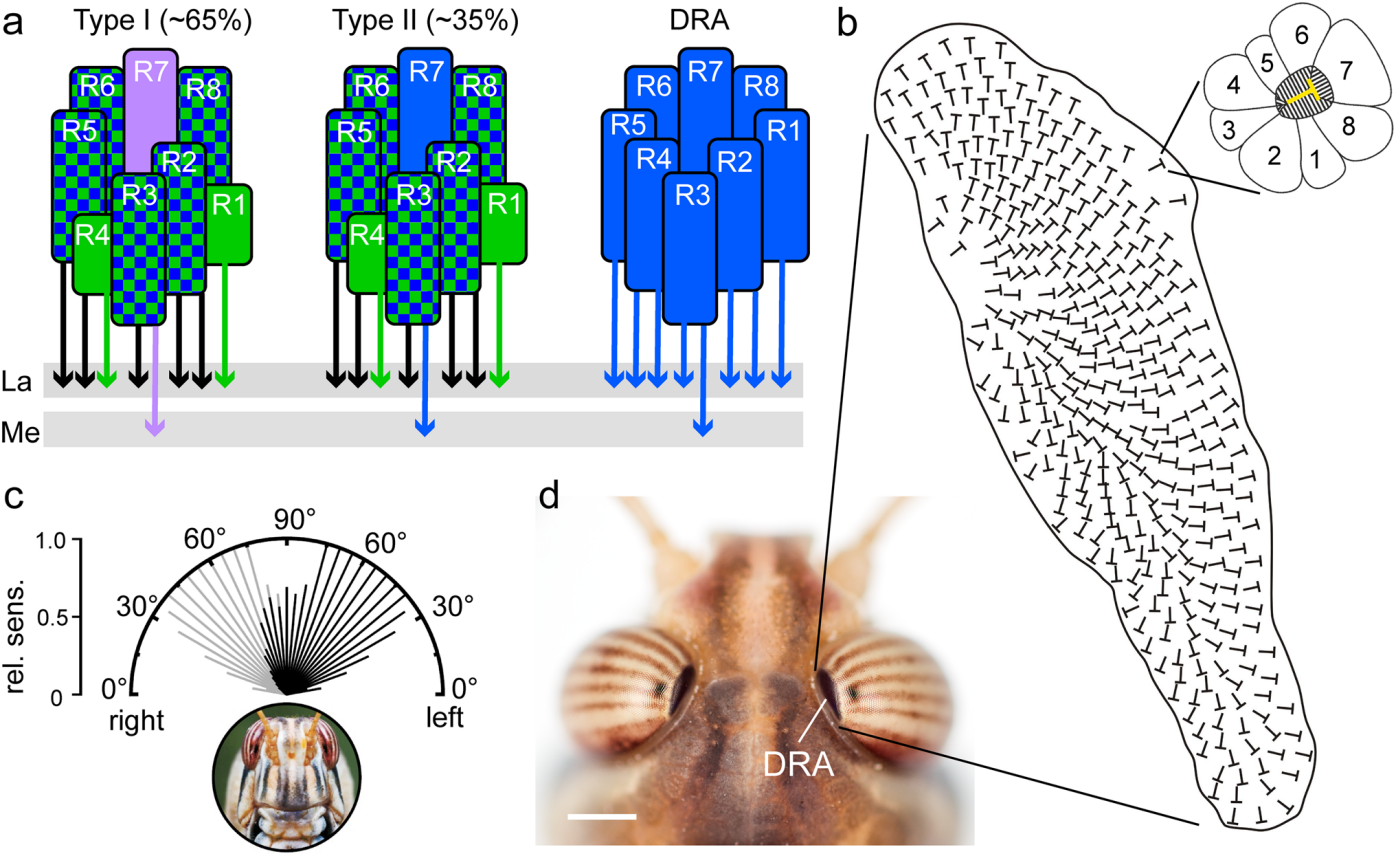
Ommatidial subtypes in the compound eye of the desert locust. **a** Photoreceptor arrangement in type I and type II ommatidia of the main eye and in dorsal rim area (DRA) ommatidia. In main eye ommatidia, R2, R3, R5, R6, and R8 ommatidia contain blue and green opsin and R1 and R4, green opsin. R7 photoreceptors contain either UV opsin (type I ommatidia) or blue opsin (type II ommatidia). Both ommatidia types are stochastically distributed in the main retina with type I ommatidia constituting about 65% and type II ommatidia, 35% of all ommatidia. In DRA ommatidia, all photoreceptors contain blue opsin. Throughout the eye photoreceptor axons of R7 terminate in the medulla, all others in the lamina. **b** Spatial arrangement of ommatidia in the DRA. The T-shaped symbols indicate the orthogonal arrangement of microvilli within ommatidia as illustrated in the enlarged ommatidium. 1–8, photoreceptors R1-R8. **c** Visual field of the DRA along the transverse axis of the locust. Black bars indicate the relative sensitivity (rel. sens.) of pooled photoreceptors of the right DRA and gray bars, relative sensitivity of photoreceptors of the left DRA. **d** Dorsal view on the locust’s head illustrating different pigmentation of the main eye and DRA. Scale bar = 1 mm. **a** From Schmeling et al. (2014), **b** from Homberg (2004), **c** from Schmeling et al. (2015), **d** photograph by Erich Staudacher

Each compound eye of the desert locust comprises about 9400 ommatidia (Bernard 1937). A small dorsal rim area (DRA) of 370–390 ommatidia is easily distinguished from the rest of the eye by darker pigmentation and irregular facet shape (Fig. 2; Homberg and Paech 2002). Each ommatidium consists of eight photoreceptor cells, termed R1-R8 (Fig. 2a). cDNA cloning revealed three opsin genes in the compound eye, a UV-type, a blue-type and a long-wavelength opsin (Schmeling et al. 2014). In situ hybridization showed that these are distributed differently in three types of ommatidia (Fig. 2a). The main part of the eye contains two stochastically distributed types of ommatidia. In both types the large photoreceptors R2, R3, R5, R6 and R8 co-express blue and green opsin, two proximal receptors R1 and R4 express green opsin and the receptor R7 expresses either UV opsin (type I ommatidium) or blue opsin (type II ommatidia). Ommatidia in the DRA, in contrast, express exclusively blue opsin in all photoreceptors (DRA ommatidia). Electrophysiological experiments support the in situ expression studies (Schmeling et al. 2014) and showed peak sensitivities at 441 nm (blue) in DRA photoreceptors and at 339 nm (UV), and 514 nm (green) in the main eye. Recordings from most photoreceptors in the main eye revealed broad spectral tuning in the green and blue range, likely resulting from co-expression of blue and green opsins. The acceptance angle – the half-width of angular space with at least 50% maximum response – of photoreceptors in the main retina is about 2°. Both eyes together provide a 360° space of vision in all directions, with 15–20° binocular overlap in the frontal equatorial field and only a slight restriction caudally below the equator (Krapp and Gabbiani 2005). The main eyes are, therefore, well suited to mediate compass coding relative to direct sunlight and the intensity and chromatic contrast across the sky.

**Fig. 3:**
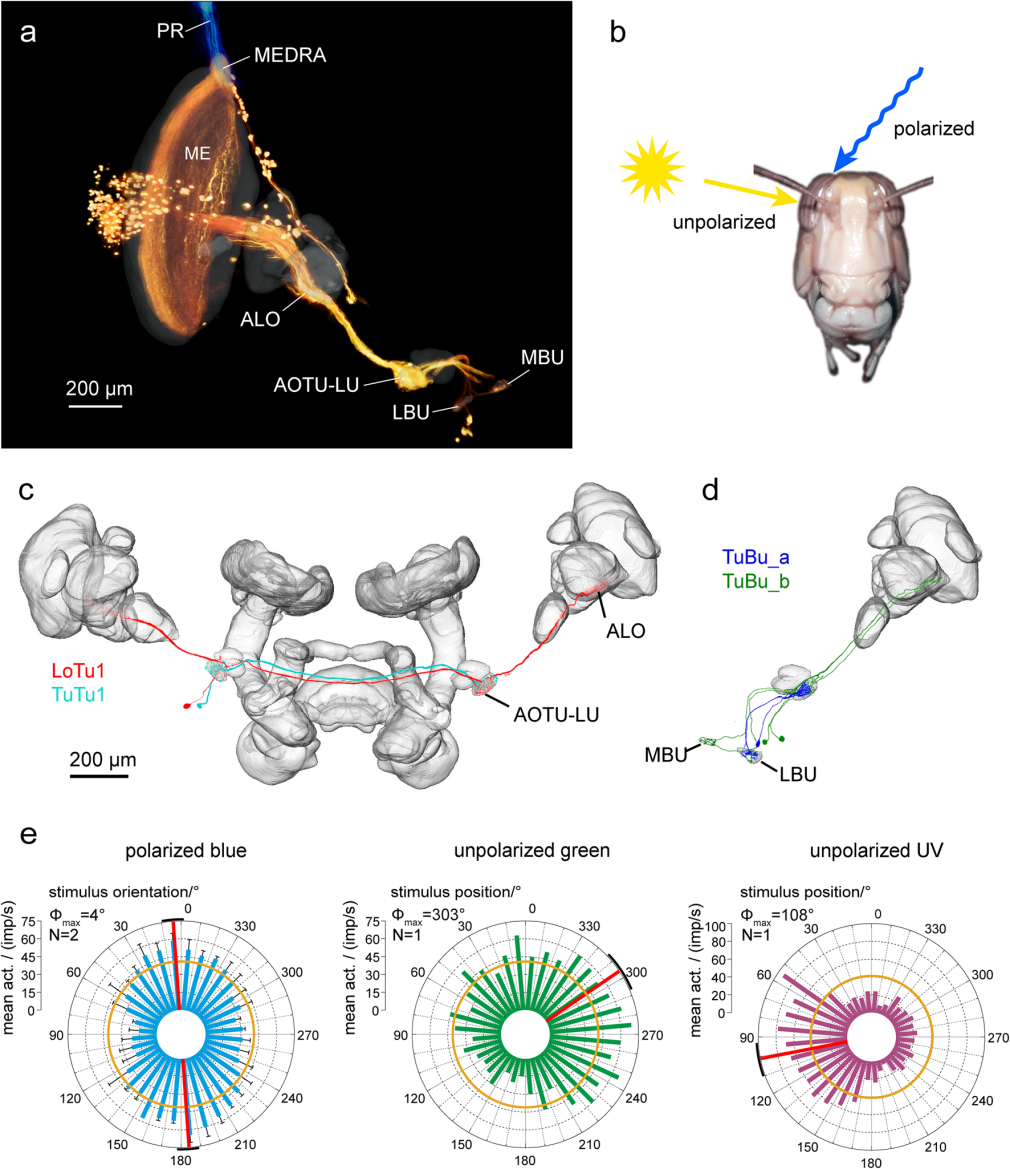
Sky compass neurons of the optic lobe and the anterior optic tubercle. **a** Tracer injection into the lower unit of the anterior optic tubercle (AOTU-LU) reveals projections of TuBu neurons to the bulbs (LBU, lateral bulb, MBU, medial bulb) and transmedulla neurons in the optic lobe. Transmedulla neurons branch in the dorsal rim area of the medulla (MEDRA), where they overlap with photoreceptor terminals (PR) from the dorsal rim area (blue), additionally labeled by tracer injection into the dorsal rim area. The unbranched neurites of the transmedulla neurons run vertically through the medulla (ME) and, as a population, cover the entire anterior–posterior extent of the neuropil. **b** Transmedulla neurons receive polarization information through the dorsal rim area and unpolarized light information through the main part of the compound eye. **c** LoTu1 and TuTu1 neurons connect the AOTU-LU of both brain hemispheres. **d** Two types of TuBu neurons connect the AOTU-LU to the bulbs. **e** Circular plots of physiological responses of a TuTu1 neuron to dorsally presented polarized light (left), an unpolarized green light spot (simulated sun, middle) and an unpolarized ultraviolet light spot (right). Spiking activity is plotted in 10° bins, black T-bars indicate SD (if number of stimulus presentations (N) > 1), orange circles indicate background activity shown in impulses (imp) s^−1^. Φ_max_ indicates the preferred angle of polarization or preferred azimuth for unpolarized light spot when the Rayleigh test indicated directedness. Φ_max_ and its 95% confidence interval are indicated by a red line and a black arc, respectively. ALO, anterior lobe of the lobula complex. **a** After el Jundi et al. (2011), **e** after Pfeiffer and Homberg (2007)

While the main retina allows for image formation, object detection, and color vision, photoreceptors in the DRA are specialized for signaling polarization angles. The microvilli of each DRA photoreceptor are highly aligned resulting in high polarization sensitivity values of up to 30, i.e. the likelihood of absorption for light that is polarized in parallel to the microvilli is 30 times higher than for light that is polarized perpendicularly (Schmeling et al. 2014). In each ommatidium R1, R2, R5, R6 and R8 have microvilli in parallel and at 90° to the microvilli of R7, while the microvilli of R3 and R4 are short and poorly aligned (Fig. 2b, inset). This orthogonal arrangement slightly rotates in adjacent ommatidia so that microvilli orientations and thus, preferred angles of polarization are represented in a fan-like arrangement across the DRA, similar to the situation in other insects (Fig. 2b; Homberg and Paech 2002). Irregularities in the facets, pore canals, lack of screening pigment between neighboring ommatidia, and large rhabdom cross-sectional area result in large acceptance angles of about 33° with considerable overlap of the receptive fields of adjacent ommatidia (Homberg and Paech 2002; Schmeling et al. 2015). Therefore, the DRA is not suited for image formation. The receptive field of the DRA covers more than 100° in anterior-posterior axis and about 80° along the transverse axis (Fig. 2c). Combining both eyes, the DRAs are, thus, sensitive to polarization angle throughout the entire sky. Interestingly, and similar to other insect species, the DRA does not face the zenith, but the optical axes of photoreceptors point contralaterally with a center at about 30° contralaterally from the zenith (Figs. 2c, 3b). This arrangement is ideally suited to integrate direct signals on the sun position with strong sky polarization cues from the anti-solar hemisphere of the sky in the same optic lobe.

Behavioral experiments on tethered locusts showed that polarotaxis to a large rotating polarizer above the animal is exclusively mediated by the DRA (Mappes and Homberg 2004) and, likewise, polarization sensitivity in sky-compass neurons of the CX is lost after painting both DRAs black (see below). Photoreceptors in the main eye have low polarization sensitivity (PS values 1.3–3.8) and are, therefore, not completely insensitive to the angle of polarization. This might be used to enhance contrast in the detection of bodies of water (Shashar et al. 2005), or light reflected from objects as shown in other insects (Heinloth et al. 2018). Accordingly, neurons in the optic lobe and central brain distinct from those of the sky-compass system are sensitive to these non-celestial polarization cues provided by the main eye (Beetz et al. 2016; Beck et al. 2022).

Throughout the eye, axonal projections of R7 photoreceptors extend into the medulla, while all other photoreceptors terminate in the lamina (Schmeling et al. 2015; Fig. 2a). Lamina and medulla areas targeted by DRA photoreceptors are clearly distinct from the rest of both neuropils (Homberg and Paech 2002). These areas are much simpler organized than the rest of the lamina and medulla, including lack of layering and overall reduced volume, suggesting that evaluation of polarization angle is their sole task. Retinotopic projections of DRA photoreceptors are, nevertheless, maintained in both accessory neuropils (Schmeling et al. 2015).

## Signatures of peripheral signal processing

Immunostaining against GABA shows that the DRAs of the lamina and the medulla are connected through GABAergic, and therefore possibly inhibitory, processes (Homberg and Paech 2002). Most polarization-sensitive neurons in insects show polarization opponency, i.e. they are maximally activated by their preferred angle of polarization (AoP) and maximally inhibited by the perpendicular AoP (Vitzthum et al. 2002). Such antagonistic responses could be established at the level of the medulla through the convergence of photoreceptors and GABAergic interneurons that receive input in the lamina. The dorsal rim area of the medulla is connected to the lower unit of the anterior optic tubercle (AOTU-LU) through transmedulla neurons (TM neurons; Homberg et al. 2003; el Jundi et al. 2011), which are called MeTu or MeTu-DRA neurons in *Drosophila* (Hardcastle et al. 2021; Kind et al. 2021; Table 1). In the medulla, these neurons consist of a thin neurite that runs dorsoventrally within the serpentine layer, and sends off a centripetal projection with ramifications in the anterior lobe of the lobula (ALO) and the AOTU-LU (Fig. 3a). The population of these dorsoventral line-like neurites occupies the entire anterior–posterior extent of the medulla and therefore likely the entire receptive field of the compound eye. While no physiological data from these neurons exists, their morphology suggests that they integrate polarized-light information from the DRA and unpolarized visual cues from the main retina. Because of the retinotopic organization of the medulla, the receptive field of the line tangential neurons obtained from photoreceptors in the main eye might have the shape of a vertical line. The population of such receptive fields could, therefore, tile the visual space around the animal, such that each neuron in the population responds to the sun when it is at a specific horizontal angle (azimuth) with respect to the animal’s head. The dorsoventrally elongated shape of the receptive field likely makes the response independent of the elevation of the sun. At the same time, polarized light information can be perceived from the opposite hemisphere, due to the tilt of the DRA’s receptive field (Fig. 3b). The TM neurons are, therefore, anatomically suited to signal head direction with respect to the solar azimuth, derive this information from either direct sunlight or the polarization pattern, and pass it on to the anterior lobe of the lobula and the anterior optic tubercle.

**Table 1:** Neuronal cell types involved in sky compass coding in the desert locust (*Schistocerca gregaria*) and corresponding cell types in the fruit fly (*Drosophila melanogaster*)

*S. gregaria*	References and notes	*D. melanogaster*	References and notes
CL1a	Heinze and Homberg (2008)	E-PG, EPG	Turner-Evans et al. (2017); Hulse et al. (2021)
CL1b, CL1d	Heinze and Homberg (2008, 2009)	P-EG, PEG	Wolff and Rubin (2018); Hulse et al. (2021)
CL1c	Heinze and Homberg (2008); lacks projections to gall	*unknown*	
CL2	Müller et al. (1997)	P-EN, PEN; two subtypes	Turner-Evans et al. (2017); Hulse et al. (2021)
CP1	Vitzthum et al. (2002)	*unknown*	
CP2	Vitzthum et al. (2002)	*unknown*	
CPU1, several subtypes	Heinze and Homberg (2008)	PFL1, PFL3	Hulse et al. (2021)
CPU2	Heinze and Homberg (2008)	PFL2	Hulse et al. (2021)
CPU4	Heinze and Homberg (2008)	PFN	Hulse et al. (2021)
LAL-LAL2	Hensgen et al. (2021a)	WL-L, WLL	Franconville et al. (2018); Hulse et al. (2021)
LoTu1	Vitzthum et al. (2002); bilateral projections in lobula	*unknown*	
pTuTu	el Jundi and Homberg (2010)	*unknown*	posterior optic tubercle not present in *Drosophila*
TB1	Heinze and Homberg (2007)	Δ7	Wolff and Rubin (2018); Δ7 neurons lack projections to the posterior optic tubercle
TB2	Heinze and Homberg (2009)	Δ7 subtype	Wolff and Rubin (2018)
TB3	Pegel et al. (2018)	P_6-8_-P_9_	Wolff and Rubin (2018); innervated columns differ from those in TB3
TB7	von Hadeln et al. (2020)	Sps-P, SpsP	Wolff et al. (2015); Wolff and Rubin (2018); Hulse et al. (2021)
TL1	Müller et al. (1997)	ExR4 (ExR6)	Omoto et al. (2018); Hulse et al. (2021); wide arborizations of ExR6 in lateral complex unlike those of TL1
TL2	Müller et al. (1997)	ER2	Hulse et al. (2021)
TL3	Müller et al. (1997)	ER3	Hardcastle et al. (2021); in contrast to TL3 not sensitive to the angle of polarization
TL4	Müller et al. (1997)	ER1 (ER3a?)	Hulse et al. (2021)
TL5	Müller et al. (1997)	ExR2	Omoto et al. (2018); in contrast to TL5 no ramifications in the PB
TM	Homberg et al. (2003)	MeTu-DRA, MeTu	Kind et al. (2021), Hardcastle et al. (2021)
TNL	this study	GLNO	Hulse et al. (2021)
TuLAL1a, TuBu_a	Pfeiffer et al. (2005); this study	TuBu, several subtypes	Hulse et al. (2021); Hardcastle et al. (2021)
TuLAL1b, TuBu_b	Pfeiffer et al. (2005); this study	*unknown*	no TuBu neurons in *Drosophila* with ramifications in the lobula
TuTu1	Homberg et al. (2003)	TuTu	Hardcastle et al. (2021)

In the medulla, five types of wide-field neuron, branching in the same layer as the TM neurons described above, are responsive to both the AoP and the azimuth of a light spot in intracellular recordings (el Jundi et al. 2011). Beyond their extensive branches in the serpentine layer of the medulla, most of these neurons have ramifications in the dorsal rim area of the medulla, the accessory medulla or both. The accessory medulla is the circadian pacemaker in a number of insects, therefore, these neurons could either provide time information to the compass system or feed information into the circadian system. While no electron microscopic data from these neurons is available, the structure of the ramifications suggests that both directions of information flow are realized. However, the specific function of these neurons is not known to date.

The next neuropil within the sky-compass pathway is layer 1 of the ALO. TM neurons that project to the AOTU-LU branch here, as well as heterolateral neurons of the AOTU-LU (LoTu1 neurons; Fig. 3c). The anterior lobe of the lobula is connected to the AOTU-LU through the anterior optic tract (Homberg et al. 2003). Lesion experiments on tethered flying locusts have shown, that polarotactic behavior is mediated exclusively through the anterior optic tract (Mappes and Homberg 2007). LoTu1 neurons seem to be missing in *Drosophila* (see Table 1).

The two heterolateral LoTu1 neurons receive input from the ipsilateral eye and provide contralateral output (Pfeiffer et al. 2005). Therefore, they establish an interhemispheric information exchange at the level of the lobula and the AOTU-LU. At the level of the AOTU-LU this interhemispheric exchange is further strengthened by two TuTu1 neurons per hemisphere, which connect the AOTU-LUs. Both LoTu1 and TuTu1 neurons show complex visual responses to both polarized and unpolarized light (Fig. 3e; Kinoshita et al. 2007; Pfeiffer and Homberg 2007; Pfeiffer et al. 2011; el Jundi and Homberg 2012). Dorsally presented polarized light leads to maximum activity at a specific AoP and to a minimum of activity at the perpendicular angle. Unpolarized light spots influence the activity based on their azimuth but also based on their wavelength. For example, in TuTu1 neurons a green light spot leads to excitation at a certain azimuth but to inhibition from the opposite side, while an ultraviolet light spot will have opposite effects (Pfeiffer and Homberg 2007). Therefore, these neurons display color opponency and spatial opponency. This double opponency could be the result of contralateral mutual inhibition, a common motif in neural circuits to amplify small sensory differences. Connectomics data from *Drosophila* confirm contralateral connections between TuTu neurons (Scheffer et al. 2020; Table 1). Functionally this is likely to help the animals to know whether the sun is to the right or the left – based solely on the spectral gradient of the sky. Support for this idea can be found in behavioral experiments. Honeybees responded to a green light spot as if it were the sun and to ultraviolet light spots as if they were lying in the antisolar hemisphere (Rossel and Wehner 1984). The integration of polarized, azimuthal and chromatic cues in the same neurons bears the potential for ambiguous signaling to downstream neurons. As these neurons are part of a system that provides a neuronal reference between sky-compass cues and the heading of the animal, it is assumed that they code for solar azimuth, but are able to derive this information from any of the available sources. Since the relationship between AoP and solar azimuth changes with solar elevation for the parts of the sky that do not lie along the solar meridian, this requires some kind of compensation. Indeed, a gradual change in the relationship between preferred solar azimuth and preferred AoP over the day has been shown for these neurons (Pfeiffer and Homberg 2007). This change is in accordance with the changes of AoP in certain areas of the sky. The AOTU-LU connects to the bulbs, the major input stations of the central complex (CX) orientation network via two types of tubercle-bulb neurons (TuBu_a and TuBu_b neurons, previously termed TuLAL1a and TuLAL1b; Fig. 3d; Pfeiffer et al. 2005).

## Multisensory integration and binocular convergence in the central complex

The heart of the neural network integrating celestial signals into an internal head-centered compass lies in the CX. Work, largely in *Drosophila*, suggests that celestial signals are combined in the CX with other spatially relevant information such as wind direction signals, internal turn-related signals, and optic flow-field information signaling travelling distance and turns (Pfeiffer and Homberg 2014; Honkanen et al. 2019; Heinze 2017; Green and Maimon 2018; Fisher 2022). The resulting heading signal is offset against an internal goal (Green et al. 2019), transformed into a world-centered coordinate system (Lyu et al. 2022; Lu et al. 2022), and integrated into steering commands in bilaterally descending neurons (Träger and Homberg 2011; Rayshubskiy et al. 2020), thereby providing the neural basis for vector-based navigation and path integration (Lyu et al. 2022; Lu et al. 2022). Studies in locusts have largely focused on analyzing sky compass representation in the CX, but the neural elements underlying additional sensory input and vector transformations are largely conserved suggesting similar spatial computations across insects.

**Fig. 4:**
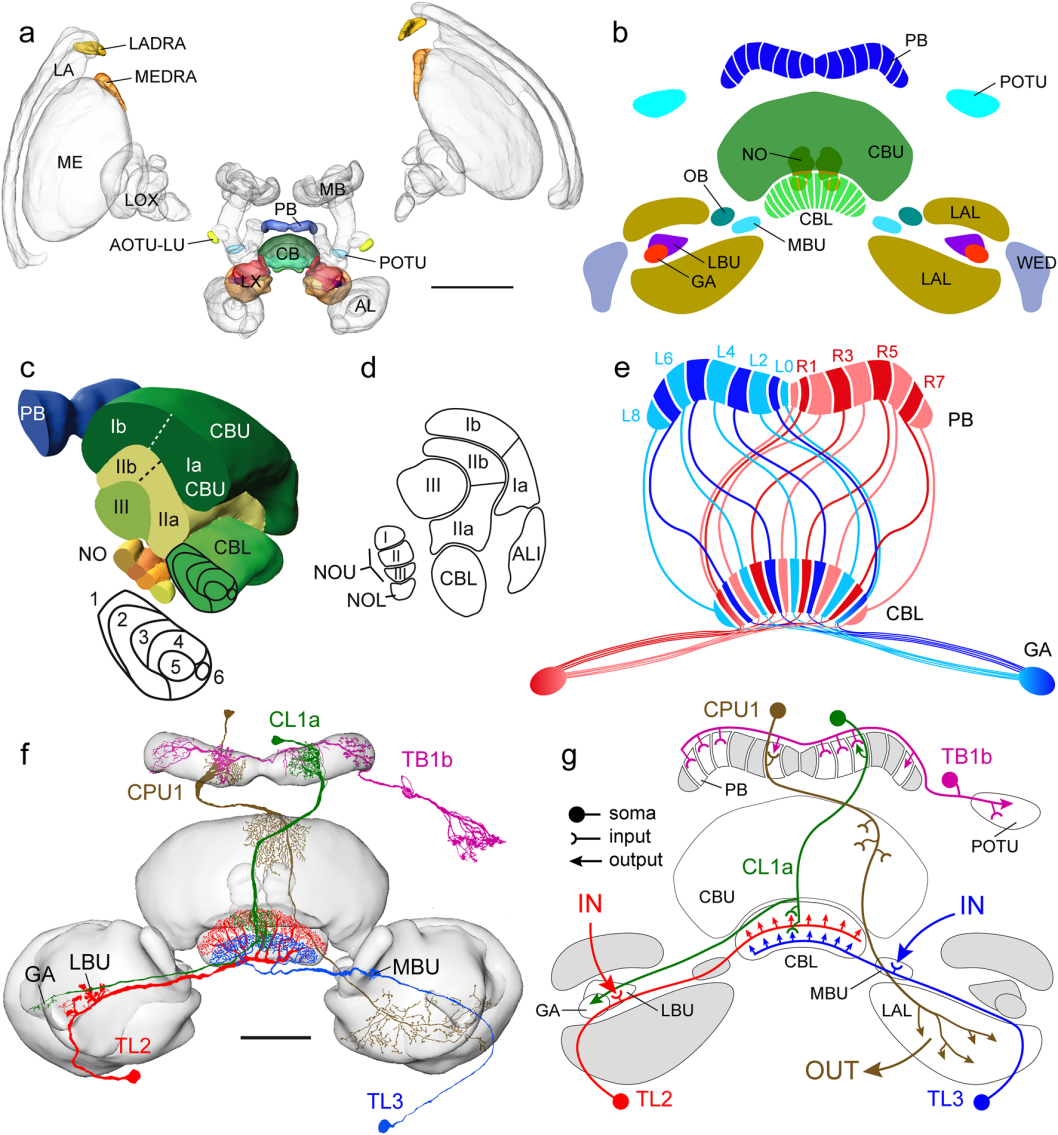
Organization of the locust central complex. **a** 3D reconstruction of the locust brain with neuropils involved in sky polarization vision illustrated in color. **b** Schematic illustration of the central complex and prominent accessory neuropils. **c** Oblique view of a sagittal section through the central complex illustrating subunits and layers. **d** Schematic sagittal section illustrating layers of the upper division of the central body (CBU) and the subunits of the noduli. **e** Connectivity scheme of a system of 18 CL1a neurons connecting columns of the lower division of the central body (CBL) to columns of the protocerebral bridge (PB) and to the gall (GA). Axonal fibers from even-numbered columns of the PB (light blue, light red) take a slightly different course to the GA than axons from odd-numbered columns. **f,g** Major types of central-complex neurons involved in sky-compass coding. **f** Reconstruction of neurons embedded into semi-transparent neuropils; **g** proposed information flow through the CX compass network. Large red and blue arrows indicate input (IN) from TuBu- to TL neurons, brown arrow indicates output (OUT) from CPU neurons to other brain areas and descending pathways. AL, antennal lobe; ALI, anterior lip; AOTU-LU, lower unit of the anterior optic tubercle; CB, central body; CBL, lower division of the CB, consisting of layers 1–6; CBU, upper division of the CB with layers Ia, Ib, IIa, IIb, and III; CL1a, type 1a columnar neuron of the CBL; CPU1, type 1 columnar neuron of the PB and CBU; GA, gall; L0-L8, columns L0-L8 in the left hemisphere of the PB; LA, lamina, LADRA, dorsal rim area of the LA; LAL, lateral accessory lobe; LBU, lateral bulb; LOX, lobula complex; LX, lateral complex; MB, mushroom body; MBU, medial bulb; ME, medulla; MEDRA, dorsal rim area of the ME; NO, nodulus; NOL, lower unit of the nodulus, NOU, upper unit of the nodulus with layers I, II and III; OB, ovoid body; POTU, posterior optic tubercle; R1-R7, columns R1-R7 in the right hemisphere of the PB; TB1b, type 1b tangential neuron of the PB; TL2/3, type 2/3 tangential neuron of the CBL. Scale bar = 500 µm (a), 100 µm (c). **c,d**, Adapted from von Hadeln et al. (2020); **e** based on data from Heinze and Homberg (2008) and Hensgen et al. (2022)

In the locust, like in other insects studied, the CX is a series of interconnected neuropils in the center of the insect brain. It consists of the upper (CBU) and lower (CBL) division of the central body, also termed fan-shaped body and ellipsoid body in flies and other species, the protocerebral bridge (PB), and a pair of globular-shaped noduli (Fig. 4a-c). The PB and CB are organized into linear arrays of vertical columns and the CBU and CBL, in addition, into a series of layers (Fig. 4b-e). The noduli are subdivided into upper and lower units (Fig. 4c,d) with the upper units organized into three distinct layers (Heinze and Homberg 2008). The PB and CB layers are interconnected through topographic chiasmal projections across the brain midline (Fig. 4e; Williams 1975; Müller et al. 1997; Heinze and Homberg 2008). Neurons of the CX provide prominent and partly topographically organized connections to neuropils of the adjacent lateral complex (LX), including the lateral accessory lobe (LAL), the lateral and medial bulb, the gall, and the ovoid body as well as the adjacent wedge of the brain (Fig. 4b).

**Fig. 5:**
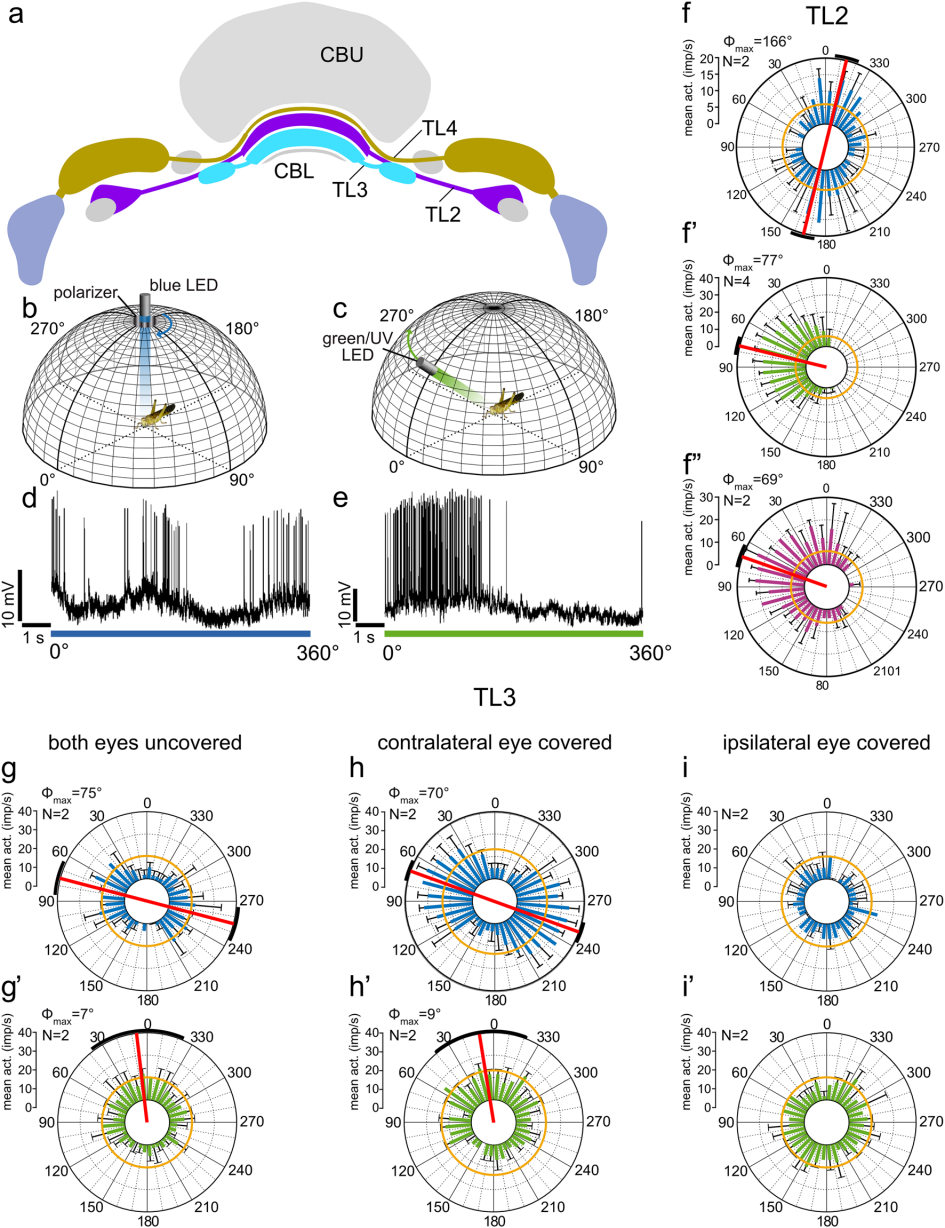
Sky compass signaling at the input stage to the central complex. **a** TL2, TL3 and TL4 neurons provide layer-specific input to the lower division of the central body (CBL). **b,c** Stimulation of the locust from the zenithal direction with blue light passing through a rotating polarizer (**b**) and with an unpolarized green (or UV) light moved at an elevation of 45° around the head of the locust (**c**). **d,e** Spike trains from a TL2 neuron during 360° rotation of the polarizer (**d**) and 360° circular movement of the unpolarized green LED around the locust’s head (**e**). **f-f’’** Circular plots from that neuron from 2 to 4 stimulations (N) with polarized blue light (**f**), an unpolarized green (simulated sun) (**f’**) and UV (**f’’**) light spot. Spiking activity is plotted in 10° bins, black T-bars indicate SD, orange circles indicate background activity shown in impulses (imp) s^−1^. Φ_max_, illustrated by a red line and a black arc (95% confidence interval) indicates preferred angle of polarization or preferred azimuth for unpolarized light spot when the Rayleigh test indicated directedness. Modified from Pegel et al. (2018). **g-i’** Circular plots of neural activity of a TL3 neuron innervating the medial bulb to a rotating zenithal polarizer (**g,h,i**) and a green (simulated sun) light spot (**g’,h’,i’**) moved in a circular path around the head of the animal as illustrated in **b** and **c**. **g-g’** Both eyes are uncovered, **h–h’** the contralateral eye is covered with black paint, **i-i’** paint on the contralateral eye removed and ipsilateral eye painted black. Preferred orientations (Φ_max_) of the neuron are indicated by red lines and confidence intervals (95%) by the black arcs. The neuron receives input to all stimuli only through the ipsilateral eye

Sky compass signals are transferred from the medial and lateral bulb via two types of tangential neuron, termed TL2 and TL3 to the CBL (Fig. 4f,g). The dendrites of both cell types form postsynaptic core elements of microglomerular synaptic complexes in the lateral (TL2) and medial (TL3) bulb (Träger et al. 2008). Here, single terminals of TuBu neurons from the AOTU-LU (Figs. 3d, 4b,g) form large cup-shaped presynaptic endings bearing well over 100 active zones with postsynaptic processes of a dense tangle of dendrites originating from one or a few TL neurons. The functional significance of this peculiar synaptic connection is not known but, judged from a large number of contacts, likely contributes to a particularly strong and reliable synaptic connection between TuBu- and TL2 and TL3 neurons. While TL2 neurons already provide binocular input to the CBL, TL3 neurons signal sky compass cues only from the ipsilateral eye (Fig. 5g-i; Vitzthum et al. 2002; Heinze et al. 2009). Neurons target largely different layers of the CBL (Figs. 4f, 5a): most TL2 neurons invade layer 2 (subtype TL2a) and layer 3 (subtype TL2b) of the six CBL layers, while most TL3 neurons invade layer 4 and 5 (subtypes TL3a, TL3b), and the rare subtype TL3c, layers 6 and 2 (Müller et al. 1997; von Hadeln et al. 2020; Takahashi et al. 2022; Hensgen et al. 2021a). Both cell types are highly sensitive to the celestial polarization angle and the azimuth of an unpolarized light spot (Fig. 5; Pegel et al. 2018). Responses to polarized light show polarization opponency, i.e. are excitatory at the preferred AoP (Φ_max_) and inhibitory at angles orthogonal to Φ_max_ (Φ_min_) (Fig. 5f,g,h; Vitzthum et al. 2002; Pegel et al. 2018; Takahashi et al. 2022). Responses to a green and UV light spot show similar azimuth tunings consistent with the hypothesis that TL2 and TL3 neurons code for the azimuth of the brightest spot but ignore chromatic aspects. These response properties are in accordance with wavelength-independent azimuth coding in CX neurons of the monarch butterfly (Heinze and Reppert, 2011) but difficult to reconcile with UV-green color opponency found in the upstream TuTu1 and LoTu1 neurons (Fig. 3e). TL2 neurons but not TL3 neurons show prominent spatial opponency to UV and green, i.e. excitation at the preferred azimuth and strong inhibition of spiking at the anti-preferred azimuth (Fig. 5f’,f’’,g’,h’). The neurons have large, cell-specific receptive fields within the visual space above the animal (Takahashi et al. 2022). Although the preferred AoP in the zenith and preferred azimuth of the simulated sun show a roughly orthogonal orientation matching the situation in the sky (Fig. 5f-f’’; Pegel et al. 2018), a considerable mismatch of sky and sun compass signals across the sky was found in most neurons (Takahashi et al. 2022).

Another tangential cell type of the CBL, TL4 neurons, are sensitive to sky compass signals (Pegel et al. 2018). TL4 neurons have dendritic ramifications in dorsal aspects of the LAL and the laterally adjacent wedge of the brain and invade layer 1 of the CBL (Fig. 5a; von Hadeln et al. 2020; Homberg et al. 2021). A single recording from a TL4 neuron showed weak AoP preference but considerably stronger, apparently achromatic azimuthal preference to green and UV light (Pegel et al. 2018). TL4 neurons appear to be homologous to ER1 and possibly ER3a neurons in *Drosophila* (Table 1; von Hadeln et al. 2020), which encode wind direction through synaptic contact provided by a particular cell type with dendrites in the antennal mechanosensory and motor center, termed WLL (Okubo et al. 2020). Homologs to WLL, present in the locust (termed LAL-LAL2 neurons, Hensgen et al. 2021a) might, likewise, receive wind input in their deutocerebral dendrites, but no physiological recordings from these neurons have been achieved yet. Double label experiments showed that TL2 and TL3 neurons are GABA-immunoreactive and thereby likely inhibit their postsynaptic partners (Homberg et al. 1999; Takahashi et al. 2022). Judged from strong GABA labeling in layer 1 of the CBL, TL4 neurons might, likewise, be GABAergic.

## An internal sky compass in the central complex

Across insect species, the neuronal cell types involved in computations within the CX network share striking morphological similarities that often result in highly similar physiologies (Pfeiffer and Homberg 2014; Turner-Evans and Jayaraman 2016; Heinze 2017; Pisokas et al. 2020; Sayre et al. 2021). This is most impressively illustrated in Table 1 listing the corresponding cell types of the sky compass system in the locust and fruit fly. The proposed connectivities of neurons that process sky compass information in the locust CX were originally based on overlapping projections and terminal fiber specializations, such as varicosities or smooth endings (e.g. Heinze and Homberg 2007; Heinze et al. 2009). More recently, optogenetic approaches, calcium imaging combined with pharmacology, and connectomics data in *Drosophila* (Franconville et al. 2018; Scheffer et al. 2020; Hulse et al. 2021) have largely established and specified corresponding connectivities in the CX of *Drosophila*, supporting the proposed transfer of sky compass signals in the locust CX (Fig. 4g).

In the CBL of the locust TL2 and TL3 neurons form synaptic contacts with each other and with CL1-type columnar neurons connecting the CBL to the PB (Fig. 4g; Heinze et al. 2009; Homberg and Müller 2016). Four subtypes of CL1 neurons have been distinguished. All of these connect single columns of the CBL and PB in a topographic way. Three of these (CL1a, CL1b, CL1d) have additional axonal projections to the gall, possibly forming global feedback loops (see below). Fiber specializations suggest that CL1a neurons transmit signals from the CBL to the PB whereas CL1b-d neurons appear to signal in opposite direction (Heinze and Homberg 2008, 2009). Likewise, in *Drosophila* two major types of neuron connect the ellipsoid body (equivalent to CBL) with the PB, EPG neurons signaling from the ellipsoid body to the PB and PEG neurons, from the PB to the ellipsoid body (Hulse et al. 2021; Table 1). In *Drosophila*, a compass-like representation of azimuthal directions in the CX emerges in EPG neurons. Calcium imaging of EPG neurons revealed a “bump” of activity in the ellipsoid body that shifts to the right as the fly walking on a treadmill or in head-fixed flight turns left and vice versa (Seelig and Jayaraman 2015; Kim et al. 2017). The underlying mechanism of signal transmission from ER neurons targeting all columns of the ellipsoid body (equivalent to locust TL neurons) to focused bump activity in EPG neurons shows properties of a ring attractor network proposed earlier to account for head-direction coding in rodents (Green and Maimon 2018; Hulse and Jayaraman 2020; Turner-Evans et al. 2020). In short, the model proposes that GABAergic ER neurons synapsing onto EPG neurons arbitrarily lead to a least inhibited EPG neuron which maintains higher activity than all others by a winner-take-all mechanism of positive feedback and global inhibition of all other EPG neurons. Important properties of this heading representation are a persistent bump position when the fly stands still, a 360° topographic representation of azimuths in the ellipsoid body leading to a 2 × 360° representation of space in the PB, and an arbitrary and different phase between azimuth and internal bump position in each fly tested (Seelig and Jayaraman 2015) that can be changed through experimental manipulation (Kim et al. 2017).

Data based on intracellular recordings from single neurons reveal parallels but also some differences in the processing and representation of spatial directions in the desert locust CX. Judged from fiber specializations in the CBL and supported by connectomics data in *Drosophila* CL1a/EPG neurons appear to be the principal postsynaptic partner of TL2/TL3 neurons in the CBL transmitting sky compass signals from the CBL to the PB (Fig. 4e-g). In the PB signals are apparently transmitted to TB neurons providing signal transmission across the columns of the PB and, finally, to columnar neurons with ramifications in single columns of the PB. When the locust is stimulated with polarized light, CL1a neurons show polarization angle-dependent inhibition but no or little net excitation as would be expected from GABAergic signal transmission from TL2/TL3 neurons (Bockhorst and Homberg 2015a). Neurons of the PB, including TB and CPU1 neurons, in contrast, show polarization-opponency (Bockhorst and Homberg 2015a). Responses to stationary compass stimuli (green light spot, stationary polarizer) revealed cell-type dependent tonic to phasic-tonic response profiles (Vitzthum et al. 2002; Bockhorst and Homberg 2015b; Takahashi et al. 2022). Significant responses of CX neurons to a small zenithal light spot occurred down to polarization degrees of 0.05 (Hensgen et al. 2022), suggesting reliable sky compass signaling even under fully overcast skies.

Analysis of topographic compass coding across the PB columns revealed mixed results: Heinze and Homberg (2009) as well as Pegel et al. (2019) did not find a systematic topography of AoP preferences in CL1 neurons across the columns of the PB. Compass-like topographies of zenithal polarization angles were, however, demonstrated in four types of neuron downstream from CL1 neurons in the PB (Heinze and Homberg 2007; Pegel et al. 2019). TB1 tangential neurons of the PB form a system of four subtypes (TB1a-d; Beetz et al. 2015). These neurons have varicose and thus likely output terminals in the outermost column 8 (TB1a), 7 (TB1b), 6 (TB1c) or 5 (TB1d) in the ipsilateral arm of the PB and a second varicose ramification tree in one of the most inner columns of the contralateral arm of the PB (columns 1–4) that are eight columns apart from their ipsilateral innervation (Fig. 4f,g). Neighboring columns are not innervated but columns adjacent to those show fine, likely dendritic processes (Fig. 4f,g). AoP preferences in TB1 neurons differed corresponding with their columnar innervation in a linear way resulting in a topographic representation of 2 × 180° across the PB (Heinze and Homberg 2007). Three types of columnar neuron showed a similar polarotopy, including CPU1 neurons. CPU1 neurons are one of the principal output elements of the CX; they have dendritic terminals in single columns of the PB, one or two columns of the CBU and axonal projections with wide ramifications in the contralateral LAL (Fig. 4f,g). While the topographies for the columnar neurons were strikingly similar, Φ_max_ representation in the TB1 neurons, determined by the position of their varicose ramifications, was phase shifted by about 50°–140° from the values of the columnar neurons. Heinze and Homberg (2007), therefore, suggested inhibitory connections between TB1 and CPU1 neurons.

**Fig. 6:**
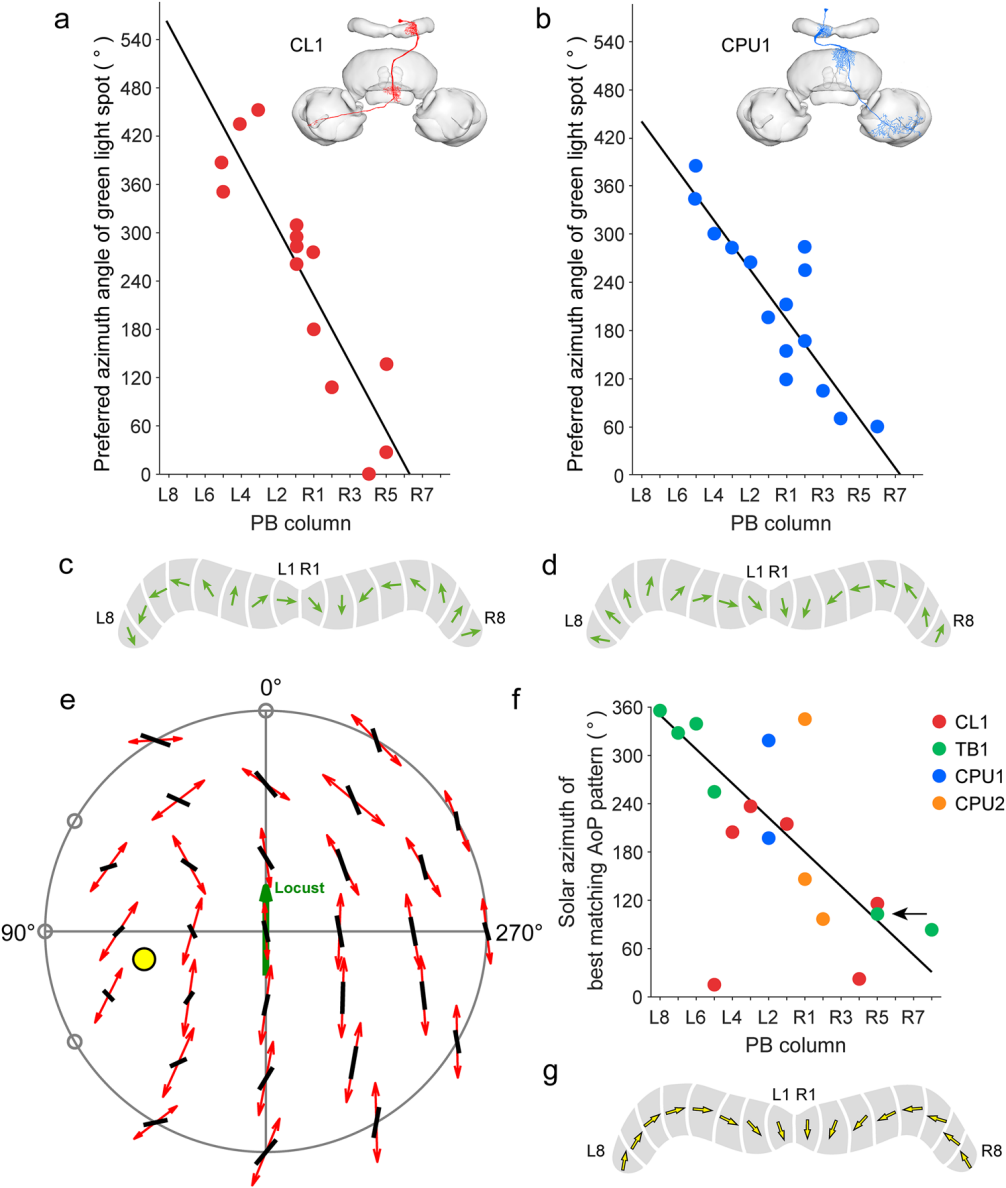
Topographic representation of sky compass coding in the protocerebral bridge (PB). **a,b** Preferred azimuth angles for a green light spot (simulated sun) of CL1 neurons (**a**) and CPU1 neurons (**b**) plotted against the columns of innervation in the PB reveal linear clockwise shifts of preferred azimuths across the PB. Circular-linear regression shows a high correlation between the innervated column of the PB and the preferred azimuth for the green light spot (**a**: *y* =−42.5*x* + 562.9; **b**: *y* =−30.9*x* + 440.0). Insets show morphologies of the two cell types. **c,d** Schematic compass topographies in the PB for CL1 neurons (**c**) and CPU1 neurons (**d**) following the regression lines in **a** and **b. e–g** Matched-filter coding of polarization patterns in the sky. **e** Top view on the pattern of preferred angles of polarization (AoPs; red double arrows) of a TB1 neuron plotted against the best-matching sky polarization pattern (black bars). This pattern corresponds to a solar position (yellow dot) at 39° elevation and 103° azimuth. Open circles along the horizon indicate non-significant AoP responses. **f** Solar azimuth of best-matching pattern of preferred AoPs plotted against the columnar innervation domains in the PB for four types of central-complex neurons. Regression analysis shows a 320° topography of solar azimuths represented in the columns of the PB (*y* =−21.3x + 350.3). Arrow points at neuron recorded in **e**. **g** Schematic compass topography in the PB following the regression line in **f**. **a-d** Based on data from Pegel et al. (2019); **e–g** from Zittrell et al. (2020)

Compass-like topographies for solar azimuth, tested through green light spots, were found in CL1 and CPU1 columnar neurons, but instead of shifting in a counterclockwise manner, like zenithal AoP preference, azimuthal tuning shifted in an opposite, clockwise manner through the columns of the PB (Fig. 6; Pegel et al. 2019). This puzzling mismatch was resolved by investigating the AoP tuning not only to zenithal stimulation but across the entire sky. Extracellular recordings revealed that AoP sensitivity of CX neurons extends across the entire sky down to the horizon (Bech et al. 2014). Moreover, AoP tuning is slightly shifted between neighboring positions in the dorsal hemisphere above the animal leading to the coding of polarization angles across the sky that match a particular sun position. Zittrell et al. (2020) repeated these studies with intracellular recordings which allowed the authors to morphologically identify the recorded neurons. That study showed that matched filter coding of sky polarization patterns occurs in TB1 tangential neurons (Fig. 6e-g) as well as in at least three types of columnar neurons (CPU1, CPU2, CL1). When using these data to plot the sun positions derived from sky polarization tuning against the columns of the PB, a clockwise solar azimuth representation resulted (Fig. 6f,g; Zittrell et al. 2020) like the solar azimuth representation in CPU1 neurons (Fig. 6b,d; Pegel et al. 2019). Interestingly, the range of azimuth coding differed between the datasets. While CL1 neurons covered an azimuth range of about 640° across the PB columns (Fig. 6a,c), CPU1 neurons represented only 460° (Fig. 6b,d), and the ensemble of CL1/TB1/CPU1/CPU2 neurons tested for solar azimuth coding through matched-filter coding of sky polarization patterns only for a range of about 320° solar azimuth (Fig. 6f,g). Reasons for these differences in azimuthal range might be statistical errors based on the small sample size used for calculating the regression lines, but might also reflect a reduction in azimuthal space from the input (CL1) to the output (CPU1 and CPU2) of the CX, even more so, as CPU neurons have not been found in the outermost columns of the PB (R7/8 and L7/8) and appear, likewise, to be absent in bees (Sayre et al. 2021).

These studies revealed two notable differences in the CX compass between the fly and the locust. First, in contrast to a 360° representation of space in EPG neurons across each arm of the PB on the fly, the range of space representation in the locust is considerably narrower and, especially in the CPU output neurons, rather covers 360° space across the entire PB, whereby the right arm of the PB encodes sun positions on the left side, and the left arm, sun positions to the right of the locust (Fig. 6d,g). Second, while the ring attractor dynamics in the fly allows for flexible representation of azimuthal space in the CX, including flexible tuning to polarized light that differs from fly to fly (Seelig and Jayaraman 2015; Hardcastle et al. 2021), data from the locust rather suggest a fixed sky-compass topography across the columns of the PB (Fig. 6). This may be achieved by topographic differences in synaptic strengths between TL2/TL3 neurons and CL1a neurons and, in the postsynaptic partners of CL1a neurons in the PB, by enforcement of topography by additional mechanisms (see below). Whether these differences reflect differences in the neuroarchitecture of the CX, such as the ventral closure of the kidney-shaped CBL (in the locust) to the toroidal ellipsoid body in the fly or is rather a consequence of different experimental designs, might be revealed by studies on further insect species and comparison of CX compass coding under restrained and freely moving conditions.

## Feedbacks and activity shifts in the sky compass

A number of cell types in addition to those displayed in Fig. 4 are involved in the sky compass network of the CX, and although their roles have not been fully established, they likely serve to stabilize the compass and aid in activity shifts during turns of the locust. Several mechanisms may operate to maintain compass signaling independent of external cues. As mentioned above, CL1b-, CL1c- and CL1d neurons follow the same connectivity scheme as CL1a neurons (Fig. 4e) but apparently signal from the PB back to the CBL (Heinze and Homberg 2009). These neurons might, like their counterparts in the fly, the PEG neurons, constitute a column-specific feedback and serve to maintain tonic activity levels signaling body orientation of the animal even in darkness (Green and Maimon 2018).

**Fig. 7:**
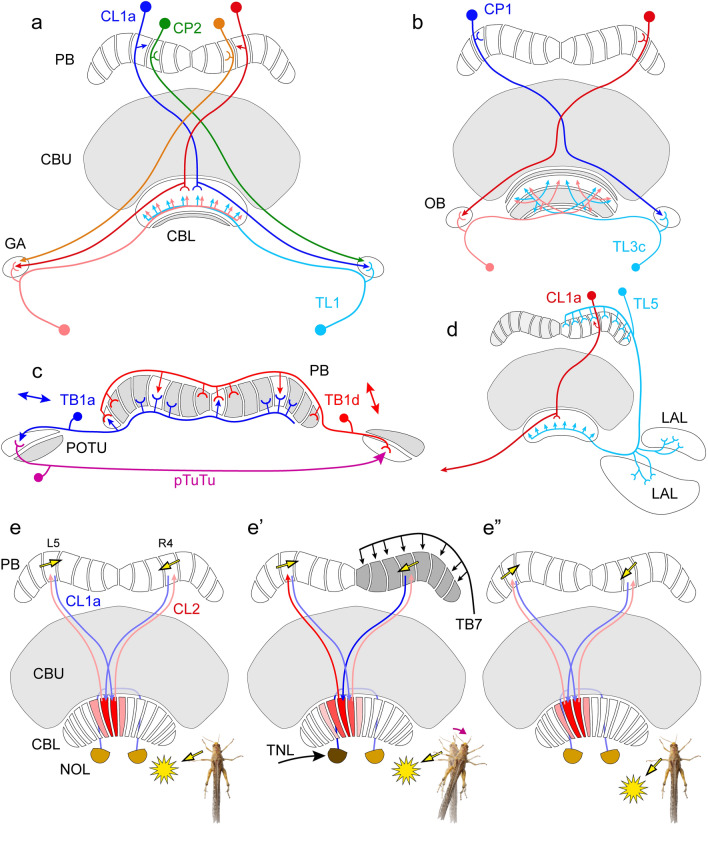
Recurrent circuits and lateral interactions in the compass network of the central complex. **a,b** Recurrent connections via the gall (GA) and ovoid body (OB). **a** The GA receives input from CL1 neurons, here illustrated by a CL1a cell, as well as CP2 columnar cells without ramifications in the central body. TL1 tangential neurons have dendrites in the GA and provide input to layers 1–5 of the CBL, thus providing a potential feedback from the PB and CBL back to the CBL. **b** A similar feedback may operate through the OBs. The OBs receive dense axonal input from CP1 columnar neurons of the PB and may contact TL3c tangential neurons innervating layers 6 and 2 of the CBL, shown in white. **c** Tangential neurons of the PB, type TB1 might interact with each other directly and via the posterior optic tubercles (POTU) to stabilize the compass topography across the PB. The image illustrates the input and output columns of a TB1a neuron in the left hemisphere and a TB1d neuron in the right hemisphere. Their tuning to polarization angle (double arrows, data from Pegel et al. 2018) is nearly orthogonal suggesting inhibitory interactions. Posterior tubercle neurons (pTuTu) connect the dorsal parts of the ipsilateral POTU to the ventral parts of the contralateral POTU. **d** TL5 tangential neurons of the CBL innervate one hemisphere of the PB and the lateral accessory lobe (LAL) and innervate all layers of the CBL, providing synchrony or feedback between these areas. **e-e’’** CL2 columnar neurons of the CBL may be involved in shifting the activity in the PB during turns of the locust. **e** CL2 neurons receive input in the PB and lower unit of a nodulus and connect to single columns of the CBL shifted by one column relative to the projection matrix of CL1a neurons. In the CBL, CL1a neurons invade at least three columns, one central column and one column to the right and left (illustrated in shades of red). Repetitive circuits involving two CL1a and two CL2 neurons might serve to maintain tonic activity during straight-line locomotion. Yellow arrows in the respective PB columns, taken from Fig. 6g, indicate opposite tuning angles, suggesting that one of the connections between CL2 and CL1a, perhaps in the PB, is inhibitory. **e’** During a right turn (magenta arrow) TNL and/or TB7 tangential neurons may be activated leading to increased activity in CL2 innervating column R4, followed by increased activity in CL1a innervating L5 in the PB, and reduced activity in CL1a innervating R4. As a consequence, columns further left in the CBL become activated shifting the activity profile in the PB to the left (**e’’**). Insets at the bottom of **e-e’’** show the orientation of the locust relative to the sun (yellow dot). **a,b** Based on Hensgen et al. (2021a); **c** based on Beetz et al. (2015)

Feedback circuits involving the gall and ovoid body might serve to balance activity levels in the right and left arms of the PB (Hensgen et al. 2021a). These recurrent loops involve two types of columnar neuron, CP1 and CP2, that innervate single columns in the PB and send axonal projections without innervation of the central body to the contralateral gall (CP2) and ovoid body (CP1), respectively. Both cell types are sensitive to sky compass signals (Vitzthum et al. 2002; Heinze and Homberg 2007; Pegel et al. 2018). In the gall, axonal terminals of CL1 and CP2 neurons overlap with dendritic ramifications of a TL neuron with a large axon, termed TL1, which invades all CBL layers except layer 6 (Fig. 7a). The TL1 neuron is moderately sensitive to sky compass signals (Vitzthum et al. 2002; Pegel et al. 2018) and is positioned to compare compass feedback from two downstream stages of compass network, the CBL (axons of CL1a) and the PB (CL1b-c; CP2). The ovoid body, a small neuropil laterally from the CBL, is densely innervated by CP1 columnar neurons of the PB. Judged from the anatomical overlap of arborizations in the ovoid body, axonal terminals of CP1 neurons are suited to contact the dendrites of a specific subtype of TL neuron, termed TL3c, which specifically targets layer 2 and 6 of the CBL (Fig. 7b), thereby forming another likely feedback from the PB back to the CBL.

Morphological overlap of pre- and postsynaptic sites of TB1 neurons of the right and left brain hemispheres are suited to maintain sky compass topography across the PB columns, partly involving the posterior optic tubercles (Fig. 7c). In *Drosophila*, mutual inhibition of Δ7 neurons, the counterparts of locust TB1 neurons (Table 1), has been demonstrated (Turner-Evans et al. 2020). In the locust, direct interactions in the PB as well as longer recurrent loops via the posterior optic tubercles may be involved (Fig. 7c). In addition to their ramifications in the PB, all TB1 neurons send apparently mixed input–output ramifications in a topographic way to one of four layers in the posterior optic tubercle, TB1a neurons connect to the most dorsal and TB1d neurons, to the most ventral layer (Beetz et al. 2015). Another type of polarization-sensitive neuron, called pTuTu, connects dorsal layers of the posterior optic tubercle on one side of the brain to ventral layers of the tubercle on the other side of the brain (el Jundi and Homberg 2010). As suggested by Beetz et al. (2015), these neurons could form a negative feedback loop between the two PB hemispheres that shape the activity profiles in the PB for topographic space coding (Fig. 7c).

Several neurons with wide ramifications in several areas of the CX compass network might serve to synchronize overall activity across the network. Among these are three different types of TCX neurons, of which at least two (TCX1 and TCX3) are sensitive to sky compass signals (Bockhorst and Homberg 2015a; von Hadeln et al. 2020). The most widely arborizing of these, TCX1, interconnects the posterior optic tubercle, the PB, the CBL, the CBU and, bilaterally, the medial and lateral bulb. Neurons with less extensive ramifications are the TL5 cells (von Hadeln et al. 2020) that connect one hemisphere of the PB and the LAL to the CBL (Fig. 7d).

Owing to topographic tuning to solar azimuth, turns of the locust should lead to a shift in the position of maximum activity across the columns of the PB. Like in *Drosophila*, this activity shift appears to be supported by the second type of CBL columnar neurons, termed CL2 (PEN in *Drosophila*; Table 1). These neurons connect single columns of the CBL and PB but have additional, likely dendritic ramifications in the lower unit of the contralateral nodulus (Fig. 7e-e’’). CL2 neurons show less robust tuning to polarized light than CL1 cells (Heinze and Homberg 2009) but, as suggested from two recordings of mirror symmetric CL2 cells, are sensitive to visual flow fields mimicking yaw rotation (Zittrell et al. 2022). Like their counterparts in *Drosophila*, the PEN neurons (Green et al. 2017; Turner-Evans et al. 2017; Table 1), the CL2 neurons had opposite directional sensitivity: the neuron innervating the right nodulus was sensitive to simulated left turns, and the neuron innervating the left nodulus, to simulated right turns of the animal. Relative to CL1 neurons, the connectivity matrix of CL2 neurons is offset by one column in the CBL and its polarity is opposite to that of CL1a neurons (Heinze and Homberg 2008). This suggests that, like in the fly, self-generated optic flow fields during turns to the left or right lead to shifts in the columnar activity peak in the PB, even if celestial compass signals are not available (Pabst et al. 2022). Input to CL2 neurons in the noduli is likely provided by tangential neurons, termed TN neurons, that specifically target the lower unit of a nodulus and are hence called TNL neurons (Homberg et al. 2013; von Hadeln et al. 2020; Table 1) but their physiology has not been investigated. A second cell type projecting from the posterior slope of the brain to one hemisphere of the PB, termed TB7, is, like its likely counterpart in *Drosophila,* the SpsP neuron (Table 1), an additional candidate for providing turn-related input (Fig. 7e’). In *Drosophila* the PEN neurons also provide turn-related signals in the dark, suggesting that they receive internal turn-related input, but this has, again not been studied in the locust.

**Fig. 8:**
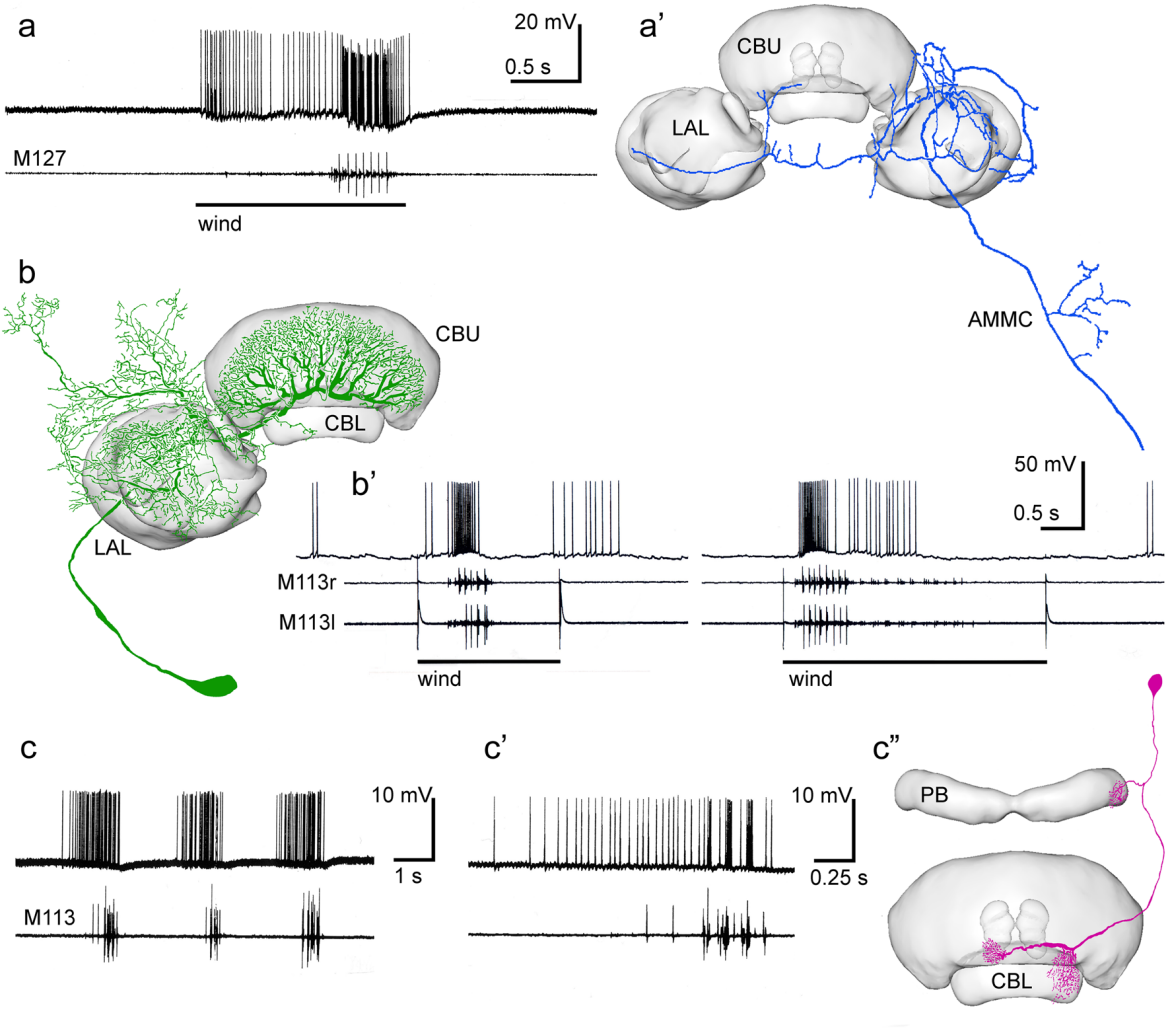
Changes in neural activity in CX neurons associated with flight. Intracellular recordings in tethered locusts combined with wire recordings from flight muscles. Legs and parts of the wings were clipped off as outlined in Homberg (1994). **a,a’** Ascending interneuron projecting to the lateral accessory lobes (LAL). Frontal wind stimulation elicits spiking activity which turns into strong bursting synchronized with flight motor activity registered by electromyographic recoding from the first basalar muscle M127. The neuron has additional projections to the antennal mechanosensory and motor center (AMMC). **b,b’** Tangential neuron of the upper division of the central body (CBU) with cell body near the vest (TU_ves_1 neuron, also termed giant fan-shaped neuron). Wind-stimulus-elicited flight motor activity recorded from the right and left tergosternal muscle (M113r, M113l) is correlated with high spiking activity. **c–c’’** Intracellular recording from a CL2 neuron. The animal showed spontaneous bouts of flight activity, which were preceded by high spiking activity. During the stationary flight, bursting activity of the neuron was correlated with flight motor activity. **c’** Enlarged first burst of activity from **c**. CBL, lower division of the central body; PB, protocerebral bridge. **a,a’** modified from Homberg (1994), **b-c’’** modified from Müller (1997) with permission

Celestial compass coding in the locust CX is modulated by unexpected visual events such as small moving objects approaching or passing by (Rosner and Homberg 2013; Bockhorst and Homberg 2015b, 2017). These stimuli lead to short disruptions of compass coding by CX neurons or to disadaptation of neural activity. Directional responses of CX compass neurons to looming stimuli might, in addition, promote directional evasive maneuvers, but the channels providing these inputs to the CX have not been elucidated so far. Analysis of the contribution of the CBU to sky compass coding and vector transformation as done in tethered walking and flying flies (Green et al. 2019; Lyu et al. 2022Lu et al. 2020) has most likely been hampered by the need to restrain the locusts during the intracellular recordings. As illustrated by panneuronal imaging experiments in *Drosophila*, the fan-shaped body, the equivalent of the locust CBU, was unresponsive in quiescent flies but became highly responsive when the animal was flying (Weir and Dickinson 2015) suggesting that the CBU is involved in flight heading. Extracellular recordings from tethered butterflies, likewise, showed that active flight can have profound effects on azimuth coding in the CX resulting in considerable phase shifts when the animal changed from rest to active flight (Beetz et al. 2022). In the locust, flight motor activity induced by frontal wind corresponds with massive increases in spiking activity in CX neurons (Fig. 8; Homberg 1994). Several cell types, including ascending neurons, as well as tangential and columnar neurons of the CX show a dramatic increase in spiking activity, partly correlated with the flight motor rhythm (Fig. 8), but how compass representations across the CX may be affected, has not been investigated. Walking activity modulates the activity of locust CX neurons at all stages of sky compass processing (Rosner et al. 2019). The effects range from changes in baseline activity in TL- and CL1 neurons to an increase in response amplitude to sky compass signals as well as to optic flow fields simulating self-movement.

Important especially for a long-range migratory animal like the desert locust is a continuous adjustment of flight direction relative to solar azimuth to maintain a constant bearing as the sun (and the polarization pattern) moves across the sky. A time-compensated sun compass orientation has been demonstrated in several insect species including the honeybee and the monarch butterfly (Lindauer 1960; Mouritsen and Frost 2002) and requires angular adjustment of flight bearing by an internal circadian clock. In many insects, notably *Drosophila* and the cockroach *Rhyparobia maderae*, the master circadian clock controlling locomotor activity rhythms is closely associated with the accessory medulla, a small neuropil at the anterior edge of the medulla (Helfrich-Förster et al. 1998; Helfrich-Förster 2005). Although a circadian pacemaker function for the accessory medulla of the locust still awaits experimental evidence, a prominent fiber fascicle specifically connects this neuropil to the posterior optic tubercle (Homberg and Würden 1997; el Jundi and Homberg 2010), where it exhibits presynaptic terminals (Held et al. 2020), offering the attractive hypothesis that time compensation may act through this pathway to shift the internal goal relative to the sky compass in the CX.

## Contribution to motor control

**Fig. 9:**
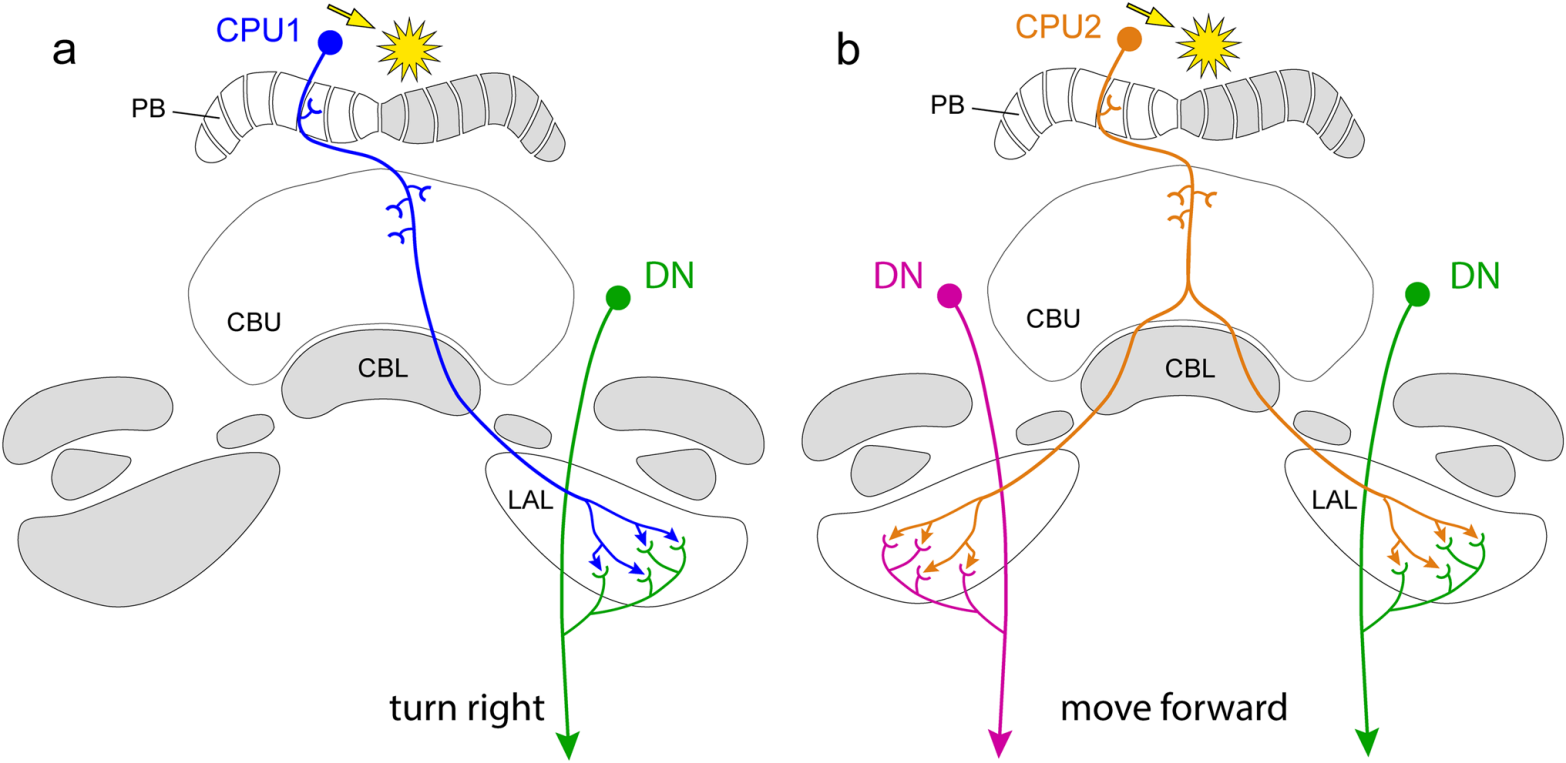
Proposed connectivity of CPU1 and CPU2 neurons with descending pathways (DN) originating in the lateral accessory lobes (LALs). **a** CPU1 neurons innervating the left hemisphere of the protocerebral bridge (PB) are highly active when the sun (arrow) is on the right side of the animal. Asymmetric activity of CPU1 neurons leads to stronger activation of descending neurons from the left than from the right lateral accessory lobe resulting in a turn of the animal. **b** Symmetric activation of descending neurons by CPU2 neurons promotes forward locomotion along a particular menotactic course relative to the solar azimuth

The most prominent output neurons of the CX are CPU1 and CPU2 neurons innervating columns in the PB and CBU and connecting these to the contralateral LAL (CPU1) or to both LALs (CPU2). Homologous neurons are present in *Drosophila* (termed PFL1/3 and PFL2, Table 1; Hulse et al. 2021) and bees (Hensgen et al. 2021b; Sayre et al. 2021). In the locust as well as other species the LALs receive numerous other inputs and are interconnected by commissural neurons (Hensgen et al. 2021a; Steinbeck et al. 2020; Namiki and Kanzaki 2016). They are directly innervated by descending neurons but also provide output to many other brain areas some of which, like the posterior slope, are likewise invaded by descending neurons (Hensgen et al. 2021a). Although physiological evidence in locusts is still missing, the connectivity pattern of CPU neurons together with data from the fly allows for hypotheses on their role in steering. While CPU2 neurons provide a balanced output to both LALs, likely promoting forward locomotion, the projections of CPU1, like their *Drosophila* counterparts, the PFL1 and 3 neurons, target the contralateral LAL only, and might, therefore, induce turns (Fig. 9). Based on the azimuth representation in the PB (Fig. 6f), CPU1 and CPU2 neurons of the right PB hemisphere are activated when the sun is to the left and CPU1 and 2 neurons of the left PB hemisphere when the sun is to the right. Both CPU types might, as has been proposed on the fly, be suited to encode the relationship between the locust’s heading direction and its internal goal direction (Hulse et al. 2021; Rayshubskiy et al. 2020). They receive compass input in the PB and, likely through their ramifications in the CBU, goal-related input derived from CBU tangential neurons such as the TU_ves_1 neuron (Fig. 8b) as well as from pontine neurons (Δh neurons in the fly) that connect individual columns across the brain midline (Hulse et al. 2021). This interaction would lead to a balanced output to both LALs, if the current heading and goal direction coincide (Fig. 9), promoting forward locomotion along a chosen azimuth or, if deviations occur, to different outputs to the right and left LAL, inducing a turn by differential activation of the right and left descending neurons.

Both ipsi- and contralateral descending neurons innervating the LAL have been identified anatomically in the locust (Homberg 1994; Hensgen et al. 2021a) but their role in steering, as demonstrated for descending neurons from the LAL in silk moths (Mishima and Kanzaki 1999), crickets (Zorović and Hedwig 2013), and *Drosophila* (Rayshubskiy et al. 2020) has not been studied. In *Drosophila*, two ipsilateral descending neurons, DNa01 and DNa02, involved in steering during walking, indeed receive major synaptic input from PFL3 neurons, thereby integrating signals from the compass in addition to inputs bypassing the CX. In the locust, steering signals from the LAL to posterior brain areas, including the posterior slope (Heinze and Homberg 2009), may likewise be involved in affecting descending pathways. Träger and Homberg (2011) identified two polarization-sensitive descending neurons with dendrites in the posterior slope of the locust. At least one of these neurons also codes for an unpolarized light spot, likely representing the sun, in an azimuth-dependent way (el Jundi et al. 2014). Recordings from these neurons showed daytime-dependent shifts in polarization angle preference (Träger and Homberg 2011), suggesting a role of these neurons in time-compensated sky compass navigation.

## Conclusions

The neural networks underlying sky compass coding have been analyzed to different degrees in crickets, locusts, bees, ants, beetles, butterflies and flies (Homberg et al. 2011; Heinze 2017; Honkanen et al. 2019; Hardcastle et al. 2021) but topographic representations of head direction coding in the CX has so far been demonstrated only in the fruit fly and the desert locust. The neural networks underlying these compasses show striking similarities but also notable differences that may be related to different ecological demands such as the need for long-range migration in the locust versus closer-range landmark-related spatial orientation in flies (Pisokas et al. 2020) but may also have an evolutionary component. As outlined in earlier paragraphs, many questions related to the use of an internal sky compass are still open and will ultimately require analyzing its operation in freely moving animals, as has been possible in rats (Lee et al., 2009), bats (Ulanovsky and Moss 2007) but also in a large cockroach (Martin et al. 2015) and praying mantis (Wosnitza et al. 2022). Nevertheless, data on sky compass networks in restrained insects, allowing researchers to individually identify the neurons under study, has widely opened the path for refined and focused analyses of navigation-based neural networks in insect brains to come.

## Data Availability

All data that support the findings of this study are available from the corresponding author.

## Abbreviations

AoP: Angle of polarization
AOTU-LU: Lower unit of the anterior optic tubercle
CBL: Lower division of the central body
CBU: Upper division of the central body
CL neurons: Columnar neurons of the protocerebral bridge and lower division of the central body
CPU neurons: Columnar neurons of the protocerebral bridge and upper division of the central body
CX: Central complex
DRA: Dorsal rim area
LAL: Lateral accessory lobe
LoTu1: Lobula-tubercle neuron 1
PB: Protocerebral bridge
TB neurons: Tangential neurons of the protocerebral bridge
TL neurons: Tangential neurons of the lower division of the central body
TM neurons: Transmedulla neurons
TNL neuron: Tangential neuron of the lower unit of the nodulus
TuBu: Tubercle-bulb neuron
TuTu1: Tubercle-tubercle neuron 1

## References

[CR1] Baker PS, Gewecke M, Cooter RJ (1984). Flight orientation of swarming *Locusta **migratoria*. Physiol Entomol.

[CR2] Bech M, Homberg U, Pfeiffer K (2014). Receptive fields of locust brain neurons are matched to polarization patterns of the sky. Curr Biol.

[CR3] Beck M, Althaus V, Pegel U, Homberg U (2022) Neurons sensitive to non-celestial polarized light in the brain of the desert locust. J Comp Physiol A (in revision) 10.1007/s00359-023-01618-w PMC10643347 36809566

[CR4] Beetz MJ, el Jundi B, Heinze S, Homberg U (2015). Topographic organization and possible function of the posterior optic tubercles in the brain of the desert locust *Schistocerca **gregaria*. J Comp Neurol.

[CR5] Beetz MJ, Pfeiffer K, Homberg U (2016). Neurons in the brain of the desert locust *Schistocerca **gregaria* sensitive to polarized light at low stimulus elevations. J Comp Physiol A.

[CR6] Beetz MJ, Kraus C, Franzke M, Dreyer D, Strube-Bloss MF, Rössler W, Warrant EJ, Merlin C, El Jundi B (2022). Flight-induced compass representation in the monarch butterfly heading network. Curr Biol.

[CR7] Bernard F (1937). Recherches sur la morphogènese des yeux composes d’arthropodes. Bull Biol Fr-Belg.

[CR8] Bockhorst T, Homberg U (2015). Amplitude and dynamics of polarization-plane signaling in the central complex of the locust brain. J Neurophysiol.

[CR9] Bockhorst T, Homberg U (2015). Compass cells in the brain of an insect are sensitive to novel events in the visual world. PLoS ONE.

[CR10] Bockhorst T, Homberg U (2017). Interaction of compass sensing and object-motion detection in the locust central complex. J Neurophysiol.

[CR11] Brines ML, Gould JL (1982). Skylight polarization patterns and animal orientation. J Exp Biol.

[CR12] Coulson KL (1988) Polarization and intensity of light in the atmosphere. A. Deepak, Hampton, VA

[CR13] Cressman K, Shroder JF, Sivanpillai R (2016). Desert locust. Biological and environmental harzards, risks, and disasters.

[CR14] el Jundi B, Homberg U (2010). Evidence for the possible existence of a second polarization-vision pathway in the locust brain. J Insect Physiol.

[CR15] el Jundi B, Homberg U (2012). Receptive field properties and intensity-response functions of polarization-sensitive neurons of the optic tubercle in gregarious and solitarious locusts. J Neurophysiol.

[CR16] el Jundi B, Pfeiffer K, Homberg U (2011). A distinct layer of the medulla integrates sky compass signals in the brain of an insect. PLoS ONE.

[CR17] el Jundi B, Pfeiffer K, Heinze S, Homberg U (2014). Integration of polarization and chromatic cues in the insect sky compass. J Comp Physiol A.

[CR18] Farrow RA, Chapman RF, Joern A (1990). Flight and migration in acridoids. Biology of grasshoppers.

[CR19] Fisher YE (2022). Flexible navigational computations in the *Drosophila* central complex. Curr Opin Neurobiol.

[CR20] Foster JJ, Smolka J, Nilsson DE, Dacke M (2018). How animals follow the stars. Proc Biol Sci.

[CR21] Franconville R, Beron C, Jayaraman V (2018) Building a functional connectome of the * Drosophila * central complex. Elife 7:e37017. 10.7554/eLife.37017 10.7554/eLife.37017 PMC6150698 30124430

[CR22] Green J, Maimon G (2018). Building a heading signal from anatomically defined neuron types in the *Drosophila* central complex. Curr Opin Neurobiol.

[CR23] Green J, Adachi A, Shah KK, Hirokawa JD, Magani PS, Maimon G (2017). A neural circuit architecture for angular integration in *Drosophila*. Nature.

[CR24] Green J, Vijayan V, Mussells Pires P, Adachi A, Maimon G (2019). A neural heading estimate is compared with an internal goal to guide oriented navigation. Nat Neurosci.

[CR25] Hardcastle BJ, Omoto JJ, Kandimalla P, Nguyen BCM, Keleş MF, Boyd NK, Hartenstein V, Frye MA (2021). A visual pathway for skylight polarization processing in *Drosophila*. Elife.

[CR26] Hegedüs R, Åkesson S, Horváth G (2007). Polarization patterns of thick clouds: overcast skies have distribution of the angle of polarization similar to that of clear skies. J Opt Soc Am A.

[CR27] Heinloth T, Uhlhorn J, Wernet MF (2018). Insect responses to linearly polarized reflections: Orphan behaviors without neural circuits. Front Cell Neurosci.

[CR28] Heinze S (2017). Unraveling the neural basis of insect navigation. Curr Opin Insect Sci.

[CR29] Heinze S, Homberg U (2007). Maplike representation of celestial *E*-vector orientations in the brain of an insect. Science.

[CR30] Heinze S, Homberg U (2008). Neuroarchitecture of the central complex of the desert locust: intrinsic and columnar neurons. J Comp Neurol.

[CR31] Heinze S, Homberg U (2009). Linking the input to the output: New sets of neurons complement the polarization vision network in the locust central complex. J Neurosci.

[CR32] Heinze S, Reppert SM (2011). Sun compass integration of skylight cues in migratory monarch butterflies. Neuron.

[CR33] Heinze S, Gotthardt S, Homberg U (2009). Transformation of polarized light information in the central complex of the locust. J Neurosci.

[CR34] Held M, Le K, Pegel U, Dersch F, Beetz MJ, Pfeiffer K, Homberg U (2020). Anatomical and ultrastructural analysis of the posterior optic tubercle in the locust *Schistocerca **gregaria*. Arthropod Struct Devel.

[CR35] Helfrich-Förster C (2005). Organization of endogenous clocks in insects. Biochem Soc Trans.

[CR36] Helfrich-Förster C, Stengl M, Homberg U (1998). Organization of the circadian system in insects. Chronobiol Int.

[CR37] Hensgen R, Göthe J, Jahn S, Hümmert S, Schneider KL, Takahashi N, Pegel U, Gotthardt S, Homberg U (2021). Organization and neural connections of the lateral complex in the brain of the desert locust. J Comp Neurol.

[CR38] Hensgen R, England L, Homberg U, Pfeiffer K (2021). Neuroarchitecture of the central complex in the brain of the honeybee: neuronal cell types. J Comp Neurol.

[CR39] Hensgen R, Dippel S, Hümmert S, Jahn S, Seyfarth J, Homberg U (2022). Myoinhibitory peptides in the central complex of the locust *Schistocerca **gregaria* and colocalization with locustatachykinin-related peptides. J Comp Neurol.

[CR40] Homberg U (1994). Flight-correlated activity changes in neurons of the lateral accessory lobes in the brain of the locust *Schistocerca **gregaria*. J Comp Physiol A.

[CR41] Homberg U (2004). In search of the sky compass in the insect brain. Naturwissenschaften.

[CR42] Homberg U (2015). Sky compass orientation in desert locusts – evidence from field and laboratory studies. Front Behav Neurosci.

[CR43] Homberg U, el Jundi B, Werner JS, Chalupa LM (2013). Polarization vision in arthropods. The new visual neurosciences.

[CR44] Homberg U, Müller M (2016). Ultrastructure of GABA- and tachykinin-immunoreactive neurons in the lower division of the central body of the desert locust. Front Behav Neurosci.

[CR45] Homberg U, Paech A (2002). Ultrastructure and orientation of ommatidia in the dorsal rim area of the locust compound eye. Arthropod Struct Devel.

[CR46] Homberg U, Würden S (1997). Movement-sensitive, polarization-sensitive, and light-sensitive neurons of the medulla and accessory medulla of the locust, *Schistocerca **gregaria*. J Comp Neurol.

[CR47] Homberg U, Vitzthum H, Müller M, Binkle U (1999). Immunocytochemistry of GABA in the central complex of the locust *Schistocerca **gregaria*: identification of immunoreactive neurons and colocalization with neuropeptides. J Comp Neurol.

[CR48] Homberg U, Hofer S, Pfeiffer K, Gebhardt S (2003). Organization and neural connections of the anterior optic tubercle in the brain of the locust, *Schistocerca **gregaria*. J Comp Neurol.

[CR49] Homberg U, Heinze S, Pfeiffer K, Kinoshita M, el Jundi B (2011). Central neural coding of sky polarization in insects. Philos Trans R Soc B.

[CR50] Homberg U, Seyfarth J, Binkle U, Monastirioti M, Alkema MJ (2013). Identification of distinct tyraminergic and octopaminergic neurons innervating the central complex of the desert locust, *Schistocerca **gregaria*. J Comp Neurol.

[CR51] Homberg U, Hensgen R, Rieber E, Seyfarth J, Kern M, Dippel S, Dircksen H, Spänig L, Kina YP (2021). Orcokinin in the central complex of the locust *Schistocerca **gregaria*: identification of immunostained neurons and colocalization with other neuroactive substances. J Comp Neurol.

[CR52] Honkanen A, Adden A, da Silva FJ, Heinze S (2019). The insect central complex and the neural basis of navigational strategies. J Exp Biol.

[CR53] Hulse BK, Jayaraman V (2020). Mechanisms underlying the neural computation of head direction. Annu Rev Neurosci.

[CR54] Hulse BK, Haberkern H, Franconville R, Turner-Evans DB, Takemura SY, Wolff T, Noorman M, Dreher M, Dan C, Parekh R, Hermundstad AM, Rubin GM, Jayaraman V (2021). A connectome of the *Drosophila* central complex reveals network motifs suitable for flexible navigation and context-dependent action selection. Elife.

[CR55] Kennedy JS (1945). Observations of the mass migration of desert locust hoppers. Trans R Entomol Soc London.

[CR56] Kennedy JS (1951). The migration of the desert locust (*Schistocerca **gregaria* FORSK.). I. The behaviour of swarms. II: A theory of long-range migrations. Phil Trans R Soc B.

[CR57] Kim SS, Rouault H, Druckmann S, Jayaraman V (2017). Ring attractor dynamics in the *Drosophila* central brain. Science.

[CR58] Kind E, Longden KD, Nern A, Zhao A, Sancer G, Flynn MA, Laughland CW, Gezahegn B, Ludwig HD, Thomson AG, Obrusnik T, Alarcón PG, Dionne H, Bock DD, Rubin GM, Reiser MB, Wernet MF (2021). Synaptic targets of photoreceptors specialized to detect color and skylight polarization in *Drosophila*. Elife.

[CR59] Kinoshita M, Pfeiffer K, Homberg U (2007). Spectral properties of identified polarized-light sensitive interneurons in the brain of the desert locust *Schistocerca **gregaria*. J Exp Biol.

[CR60] Krapp H, Gabbiani F (2005). Spatial distribution of inputs and local receptive field properties of a wide-field, looming sensitive neuron. J Neurophysiol.

[CR61] Labhart T, Meyer EP (1999). Detectors for polarized skylight in insects: a survey of ommatidial specializations in the dorsal rim area of the compound eye. Microsc Res Tech.

[CR62] Lee AK, Epsztein J, Brecht M (2009). Head-anchored whole-cell recordings in freely moving rats. Nat Protoc.

[CR63] Lindauer M (1960). Time-compensated sun orientation in bees. Cold Spring Harb Symp Quant Biol.

[CR64] Lu J, Behbahani AH, Hamburg L, Westeinde EA, Dawson PM, Lyu C, Maimon G, Dickinson MH, Druckmann S, Wilson RI (2022). Transforming representations of movement from body- to world-centric space. Nature.

[CR65] Lyu C, Abbott LF, Maimon G (2022). Building an allocentric travelling direction signal via vector computation. Nature.

[CR66] Mappes M, Homberg U (2004). Behavioral analysis of polarization vision in tethered flying locusts. J Comp Physiol A.

[CR67] Mappes M, Homberg U (2007). Surgical lesion of the anterior optic tract abolishes polarotaxis in tethered flying locusts, *Schistocerca **gregaria*. J Comp Physiol A.

[CR68] Martin JP, Guo P, Mu L, Harley CM, Ritzmann RE (2015). Central-complex control of movement in the freely walking cockroach. Curr Biol.

[CR69] Mishima T, Kanzaki R (1999). Physiological and morphological characterization of olfactory descending interneurons of the male silkworm moth, *Bombyx mori*. J Comp Physiol A.

[CR70] Mouritsen H (2000). Navigation in birds and other animals. Image Vision Comput.

[CR71] Mouritsen H, Frost BJ (2002). Virtual migration in tethered flying monarch butterflies reveals their orientation mechanisms. Proc Natl Acad Sci U S A.

[CR72] Müller M, Homberg U, Kühn A (1997). Neuroarchitecture of the lower division of the central body in the brain of the locust (*Schistocerca **gregaria*). Cell Tissue Res.

[CR73] Müller M (1997) Morphologische und funktionelle Charakterisierung der unteren Einheit des Zentralkörpers im Gehirn der Heuschrecke *Schistocerca gregaria*. Doctoral thesis, University of Regensburg

[CR74] Namiki S, Kanzaki R (2016). Comparative neuroanatomy of the lateral accessory lobe in the insect brain. Front Physiol.

[CR75] Okubo TS, Patella P, D'Alessandro I, Wilson RI (2020). A neural network for wind-guided compass navigation. Neuron.

[CR76] Omoto JJ, Nguyen BM, Kandimalla P, Lovick JK, Donlea JM, Hartenstein V (2018). Neuronal constituents and putative interactions within the *Drosophila* ellipsoid body neuropil. Front Neural Circuits.

[CR77] Pabst K, Zittrell F, Homberg U, Endres DM (2022) A model for optic flow integration in locust central-complex neurons tuned to head direction. Proc Annu Meeting Cog Science Soc 44. https://escholarship.org/uc/item/17k48481

[CR78] Pegel U, Pfeiffer K, Homberg U (2018). Integration of celestial compass cues in the central complex of the locust brain. J Exp Biol.

[CR79] Pegel U, Pfeiffer K, Zittrell F, Scholtyssek C, Homberg U (2019). Two compasses in the central complex of the locust brain. J Neurosci.

[CR80] Pener MP, Simpson SJ (2009). Locust phase polyphenism: an update. Adv Insect Physiol.

[CR81] Pfeiffer K, Homberg U (2007). Coding of azimuthal directions via time-compensated combination of celestial compass cues. Curr Biol.

[CR82] Pfeiffer K, Homberg U (2014). Organization and functional roles of the central complex in the insect brain. Annu Rev Entomol.

[CR83] Pfeiffer K, Kinoshita M, Homberg U (2005). Polarization-sensitive and light-sensitive neurons in two parallel pathways passing through the anterior optic tubercle in the locust brain. J Neurophysiol.

[CR84] Pfeiffer K, Negrello M, Homberg U (2011). Conditional perception under stimulus ambiguity: polarization- and azimuth-sensitive neurons in the locust brain are inhibited by low degrees of polarization. J Neurophysiol.

[CR85] Pisokas I, Heinze S, Webb B (2020). The head direction circuit of two insect species. Elife.

[CR86] Rayshubskiy A, Holtz SL, D’Alessandro I, Li AA, Vanderbeck QX, Haber IS, Gibb PW, Wilson RI (2020). Neural circuit mechanisms for steering control in walking *Drosophila*. BioRxiv.

[CR87] Roberts NW, Porter ML, Cronin TW (2011). The molecular basis of mechanisms underlying polarization vision. Philos Trans R Soc Lond B Biol Sci.

[CR88] Roffey J (1963). Observations on night flight in the desert locust (*Schistocerca **gregaria* Forskål). Anti-Locust Bull.

[CR89] Rosner R, Homberg U (2013). Widespread sensitivity to looming stimuli and small moving objects in the central complex of an insect brain. J Neurosci.

[CR90] Rosner R, Pegel U, Homberg U (2019). Responses of compass neurons in the locust brain to visual motion and leg motor activity. J Exp Biol.

[CR91] Rossel S, Wehner R (1984). Celestial orientation in bees: the use of spectral cues. J Comp Physiol.

[CR92] Sayre ME, Templin R, Chavez J, Kempenaers J, Heinze S (2021). A projectome of the bumblebee central complex. Elife.

[CR93] Scheffer LK, Xu CS, Januszewski M, Lu Z, Takemura SY, Hayworth KJ, Huang GB, Shinomiya K, Maitlin-Shepard J, Berg S, Clements J, Hubbard PM, Katz WT, Umayam L, Zhao T, Ackerman D, Blakely T, Bogovic J, Dolafi T, Kainmueller D, Kawase T, Khairy KA, Leavitt L, Li PH, Lindsey L, Neubarth N, Olbris DJ, Otsuna H, Trautman ET, Ito M, Bates AS, Goldammer J, Wolff T, Svirskas R, Schlegel P, Neace E, Knecht CJ, Alvarado CX, Bailey DA, Ballinger S, Borycz JA, Canino BS, Cheatham N, Cook M, Dreher M, Duclos O, Eubanks B, Fairbanks K, Finley S, Forknall N, Francis A, Hopkins GP, Joyce EM, Kim S, Kirk NA, Kovalyak J, Lauchie SA, Lohff A, Maldonado C, Manley EA, McLin S, Mooney C, Ndama M, Ogundeyi O, Okeoma N, Ordish C, Padilla N, Patrick CM, Paterson T, Phillips EE, Phillips EM, Rampally N, Ribeiro C, Robertson MK, Rymer JT, Ryan SM, Sammons M, Scott AK, Scott AL, Shinomiya A, Smith C, Smith K, Smith NL, Sobeski MA, Suleiman A, Swift J, Takemura S, Talebi I, Tarnogorska D, Tenshaw E, Tokhi T, Walsh JJ, Yang T, Horne JA, Li F, Parekh R, Rivlin PK, Jayaraman V, Costa M, Jefferis GS, Ito K, Saalfeld S, George R, Meinertzhagen IA, Rubin GM, Hess HF, Jain V, Plaza SM (2020). A connectome and analysis of the adult *Drosophila* central brain. Elife.

[CR94] Schmeling F, Wakakuwa M, Tegtmeier J, Kinoshita M, Bockhorst T, Arikawa K, Homberg U (2014). Opsin expression, physiological characterization and identification of photoreceptor cells in the dorsal rim area and main retina of the desert locust, *Schistocerca **gregaria*. J Exp Biol.

[CR95] Schmeling F, Tegtmeier J, Kinoshita M, Homberg U (2015). Photoreceptor projections and receptive fields in the dorsal rim area and main retina of the locust eye. J Comp Physiol A.

[CR96] Seelig JD, Jayaraman V (2015). Neural dynamics for landmark orientation and angular path integration. Nature.

[CR97] Shashar N, Sabbah S, Ahoni N (2005). Migrating locusts can detect polarized reflections to avoid flying over the sea. Biol Lett.

[CR98] Steinbeck F, Adden A, Graham P (2020) Connecting brain to behaviour: a role for general purpose steering circuits in insect orientation? J Exp Biol 223:jeb212332. 10.1242/jeb.212332 10.1242/jeb.212332 32161054

[CR99] Strutt, JW (1871) XV. On the light from the sky, its polarization and colour. Phil Mag 41:107–120 & 274–279.

[CR100] Takahashi N, Zittrell F, Hensgen R, Homberg U (2022). Receptive field structures for two celestial compass cues at the input stage of the central complex in the locust brain. J Exp Biol.

[CR101] Träger U, Homberg U (2011). Polarization-sensitive descending neurons in the locust: connecting the brain to thoracic ganglia. J Neurosci.

[CR102] Träger U, Wagner R, Bausenwein B, Homberg U (2008). A novel type of microglomerular synaptic complex in the polarization vision pathway of the locust brain. J Comp Neurol.

[CR103] Turner-Evans DB, Jayaraman V (2016). The insect central complex. Curr Biol.

[CR104] Turner-Evans D, Wegener S, Rouault H, Franconville R, Wolff T, Seelig JD, Druckmann S, Jayaraman V (2017). Angular velocity integration in a fly heading circuit. Elife.

[CR105] Turner-Evans DB, Jensen KT, Ali S, Paterson T, Sheridan A, Ray RP, Wolff T, Lauritzen JS, Rubin GM, Bock DD, Jayaraman V (2020). The neuroanatomical ultrastructure and function of a biological ring attractor. Neuron.

[CR106] Ugolini A, Hoelters L, Ciofini A, Paquali V, Wilckoson DC (2016). Evidence for discrete solar and lunar orientation mechanisms in the beach amphipod, *Talitrus** saltator* Montagu (Crustacea, Amphipoda). Sci Rep.

[CR107] Ulanovsky N, Moss CF (2007). Hippocampal cellular and network activity in freely moving echolocating bats. Nat Neurosci.

[CR108] Vitzthum H, Müller M, Homberg U (2002). Neurons of the central complex of the locust *Schistocerca **gregaria* are sensitive to polarized light. J Neurosci.

[CR109] von Hadeln J, Hensgen R, Bockhorst T, Rosner R, Heidasch R, Pegel U, Quintero Pérez  M, Homberg U (2020). Neuroarchitecture of the central complex of the desert locust: Tangential neurons. J Comp Neurol.

[CR110] Wehner R (1984). Astronavigation in insects. Annu Rev Entomol.

[CR111] Weir PT, Dickinson MH (2015). Functional divisions for visual processing in the central brain of flying *Drosophila*. Proc Natl Acad Sci USA.

[CR112] Williams JLD (1975). Anatomical studies of the insect central nervous system: a ground-plan of the midbrain and an introduction to the central complex in the locust, *Schistocerca **gregaria* (Orthoptera). J Zool.

[CR113] Wolff T, Rubin GM (2018). Neuroarchitecture of the *Drosophila* central complex: A catalog of nodulus and asymmetrical body neurons and a revision of the protocerebral bridge catalog. J Comp Neurol.

[CR114] Wolff T, Iyer NA, Rubin GM (2015). Neuroarchitecture and neuroanatomy of the *Drosophila* central complex: A GAL4-based dissection of protocerebral bridge neurons and circuits. J Comp Neurol.

[CR115] Wosnitza A, Martin JP, Pollack AJ, Svenson GJ, Ritzmann RE (2022). The role of central complex neurons in prey detection and tracking in the freely moving praying mantis (*Tenodera** sinensis*). Front Neural Circuits.

[CR116] Zittrell F, Pfeiffer K, Homberg U (2020). Matched-filter coding of sky polarization results in an internal sun compass in the brain of the desert locust. Proc Natl Acad Sci U S A.

[CR117] Zittrell F, Pabst K, Carlomagno E, Rosner R, Pegel U, Endres DM, Homberg U (2022) Integration of optic flow into the sky compass network in the brain of the desert locust. BioRxiv. 10.1101/2022.11.24.517785 10.3389/fncir.2023.1111310 PMC10175609 37187914

[CR118] Zorović M, Hedwig B (2013). Descending brain neurons in the cricket *Gryllus*
*bimaculatus* (de Geer): auditory responses and impact on walking. J Comp Physiol A.

